# Factors related to environmental barriers experienced by persons with and without disabilities in diverse African settings

**DOI:** 10.1371/journal.pone.0186342

**Published:** 2017-10-12

**Authors:** Surona Visagie, Arne H. Eide, Karin Dyrstad, Hasheem Mannan, Leslie Swartz, Marguerite Schneider, Gubela Mji, Alister Munthali, Mustafa Khogali, Gert van Rooy, Karl-Gerhard Hem, Malcolm MacLachlan

**Affiliations:** 1 Centre for Rehabilitation Studies, Stellenbosch University, Tygerberg, South Africa; 2 SINTEF Technology and Society, Oslo, Norway; 3 School of Nursing, Midwifery & Health Systems, University College Dublin, Dublin, Ireland; 4 Stellenbosch University, Department of Psychology, Stellenbosch, South Africa; 5 Alan J Flisher Centre for Public Mental Health, University of Cape Town, Cape Town, South Africa; 6 University of Malawi, Zomba, Malawi; 7 Afhad University for Women, Omdurman, Sudan; 8 University of Namibia, Windhoek, Namibia; 9 Department of Psychology, Maynooth University, Maynooth, Ireland; 10 Olomouc University Social Health Institute, Palacky University Olomouc, Olomouc, Czech Republic; Universita degli Studi di Perugia, ITALY

## Abstract

This paper explores differences in experienced environmental barriers between individuals with and without disabilities and the impact of additional factors on experienced environmental barriers. Data was collected in 2011–2012 by means of a two-stage cluster sampling and comprised 400–500 households in different sites in South Africa, Sudan Malawi and Namibia. Data were collected through self-report survey questionnaires. In addition to descriptive statistics and simple statistical tests a structural equation model was developed and tested. The combined file comprised 9,307 participants. The Craig Hospital Inventory of Environmental Factors was used to assess the level of environmental barriers. Transportation, the natural environment and access to health care services created the biggest barriers. An exploratory factor analysis yielded support for a one component solution for environmental barriers. A scale was constructed by adding the items together and dividing by number of items, yielding a range from one to five with five representing the highest level of environmental barriers and one the lowest. An overall mean value of 1.51 was found. Persons with disabilities scored 1.66 and persons without disabilities 1.36 (F = 466.89, p < .001). Bivariate regression analyses revealed environmental barriers to be higher among rural respondents, increasing with age and severity of disability, and lower for those with a higher level of education and with better physical and mental health. Gender had an impact only among persons without disabilities, where women report more barriers than men. Structural equation model analysis showed that socioeconomic status was significantly and negatively associated with environmental barriers. Activity limitation is significantly associated with environmental barriers when controlling for a number of other individual characteristics. Reducing barriers for the general population would go some way to reduce the impact of these for persons with activity limitations, but additional and specific adaptations will be required to ensure an inclusive society.

## Introduction

The International Classification of Functioning, Disability and Health (ICF) provides a framework for disability that incorporates environmental factors, i.e. those factors that “make up the physical, social and attitudinal environment in which people live and conduct their lives” [1:10]. Following the ICF, environmental factors can enhance participation or act as barriers and decrease participation, either increasing functioning opportunities in the former case, or restricting participation in the latter [2–7]. While the role of the environment has been widely recognised in the experience of disability [2,8,9,10], a systematic literature review by Cerniauskaite et. al. [11] showed few studies on environmental barriers in ICF related research. Articles that did report research on environmental barriers indicated that generally more environmental barriers are experienced in countries with fewer resources than in those with more resources [12]; and in rural compared to urban areas within a country [13].

Exploring the interaction of environmental barriers with ICF functioning components (at body, activity and participation levels) may contribute to identifying both equitable policy initiatives and development of individualized and appropriate public goods and services that can improve the quality of life of people with disabilities. The models utilized in the analysis draws on the ICF model and incorporates factor that represent the component of health, individual factors, environmental factors and activity limitations, and may thus contribute to further understanding the application of the ICF model. This is concurrent with the invitation from WHO to use the components in the ICF to create models that allows for analyses of different aspects of the disablement process (1:18).

This article will analyse differences in experienced environmental barriers between individuals with and without disability, and the impact of additional factors, in four different African contexts, as well as comparing the association between environmental barriers and disability across the four study settings.

### The role of the environment in participation

The ICF classifies environmental factors into five main domains. The first domain, products and technology, includes access to food, assistive devices, technology, adapted and otherwise, medication, finances and other assets. Research has shown a lack of assets [7,14,15], limited access to food, products for daily living and communication [7,14,15,16], a lack of access to private and public buildings [7,15,16,17,18], a lack of access to transport [7,15,17,19,20], and a lack of access to assistive devices as barriers for persons with disabilities [15].

The natural environment and human-made changes to the environment (e.g. terrain, climate, built environment and walkways) constitutes the second domain and has been identified as creating barriers to participation for persons with disabilities [6,7,15,16,18]. The third domain focuses on support and relationships with others (e.g. with close family and friends, people in authority, service providers and strangers). Persons with disabilities valued support from family, friends and community members and see practical assistance as a means to improve participation while a lack of assistance created severe barriers to participation [7,15,18,19]. Similarly, health care service providers were seen as a source of support that assisted facilitation of participation by some [18] and a barrier to participation by others [19].

The fourth domain, attitudes of others, has also been identified as a barrier to participation of person with disabilities [4,7,21]. The fifth and final domain encompasses services, systems and policies across a range of areas such as education, housing, health, labour, transport and basic services. In this regard Cawood & Visagie [15] identified housing, transport, communication and social services as barriers in a South African setting. Maart et al [16] added educational and labour services to the list. Inequitable policy is well established as a barrier, with the potential of becoming powerful facilitator, for participation [22] and in particular across the four countries in which the current research was conducted [23].

The ICF [1] presents functioning and its counterpart, disability, as the outcome of the interaction between a person with a health condition and the context in which that person lives. This interaction comprises both characteristics of the individual (impairments, activity limitations), and attributes of the environment (environmental factors), resulting in participation or participation restriction as an outcome. A key feature of this continuum is that all people experience difficulties in doing various activities at some point in their lives–it is not just about persons with disabilities [1]. In addition, environmental barriers are seldom experienced in isolation; it is more common for an individual to experience multiple barriers simultaneously, such as low income, access to services, belonging to a minority group and other barriers, which then have an accumulative effect on participation [7]. For example, a combination of lack of transport, inaccessible workplaces, poor education services and stigma often make it very difficult for a person with disabilities to find employment.

Environmental factors can further be categorised into those affecting everyone, both disabled and non-disabled, and those that are specifically related to various disabilities. For example, poor accessibility of public transport and difficult terrain in rural areas with poor infrastructure affect all people, but the extent to which it affects people with disabilities may be qualitatively different in increasing exclusion. Other barriers, such as access to assistive technology and stigma are specifically related to disability. Thus, and supported by the literature on disability and environmental barriers, our hypothesis is that the presence of disability/activity limitations is directly associated with environmental barriers–also when controlling for individual and contextual characteristics that are known to differ between persons with and without disabilities. Health variables, SES and Personal factors included in our model are all known to vary between persons with and without disability and may potentially influence the relationship between disability and environmental barriers.

### Study context

The study was carried out in 2011–2012 in four different sites in each of South Africa, Sudan and Malawi, and five sites in Namibia, as part of an EU funded collaborative research project on access to health care for vulnerable people in resource poor settings (Equitable). As described elsewhere [24], the selection of study sites was carried out in each country, deliberately including populations with different characteristics in order to highlight particular characteristics of each country. All sites were characterised by low resources and a combination of one or more of the following characteristics: a high proportion of internally displaced people, dispersed populations, chronic poverty and high illness burden, inequity in access to health care and other resources. Site selection did not aim to be nationally representative, but to capture specific vulnerable populations in each country. Clusters within all sites were selected on the basis of the above mentioned predefined characteristics as well as practical considerations. Further details of the sampling procedure can be found in Eide et al [24].

## Methods

Data were collected through self-report survey questionnaires (S1–S3 Files). Questions were asked in a face to face format with respondents selecting the appropriate response option for each question. Data collection was carried out by teams of interviewers led by a supervisor who checked and verified each completed questionnaire. In SA only, data were collected by means of cell phones with a built-in control system. A standard introduction was read to all respondents and oral consent was chosen due to the low rates of functional literacy in large parts of the sample.

The study population included all people living in the various study sites. As described previously [24], ta sample size of 400–500 households (HHs) per site in each country was considered sufficient. Participants (all individuals in samples household > 5 years) were identified through two-stage cluster sampling. The Washington Group Short Set of six questions [25] were applied as screening instrument and were combined to be used as a disability index in the analyses. The required number of households (400–500) with at least one member with an activity limitation was subsequently randomly sampled from the total number of screened households. Matched by age and gender, individual controls were selected within the sampled HHs.

Three different questionnaires were used, in addition to the screening questions: A household questionnaire (S1 File) and two individual questionnaires, one for individuals with disabilities (S2 File) and one for non-disabled controls (S3 File). The questionnaires all drew on previous experience with large scale studies in southern Africa [26], were adapted to the purpose of the study, and included mostly validated research instruments. The screening questions and the household questionnaire were administered to the head of the household, while the individual questionnaire was administered to individuals with and without disabilities respectively. A proxy was used for children under 14 years and for older children and adults who were unable to respond.

Of the variables in the model, Environmental barriers and Activity limitations were validated prior to the study, Possession scale was drawn from similar battery of indicators in use by Central Statistical Offices in the participating countries, while the remaining variables were straightforward and easy to understand (e.g. gender). The Research Team worked extensively in several rounds on the questionnaires in order to ensure that they were relevant for the contexts, and in all countries small-scale trial data collections were carried out prior to the main data collection to ensure comprehension.

To measure environmental barriers, the questionnaire included the Craig Hospital Inventory of Environmental Factors (CHIEF) [27]. The scale comprises 10 different environmental factors and respondents are asked how often the different items on the scale are experienced as barriers (Never = 1, Less than monthly = 2, Monthly = 3, Weekly = 4, Daily = 5). The environmental factors include services of transportation and health care, features of the natural environment, help from others at home, attitudes at home and in the community, and experiences of prejudice. Two items from the original scale were excluded due to a large number of missing observations, namely, having someone to help at school or at work and people's attitudes at school or at work. A high proportion of the missing observations were from individuals identified with a disability, reflecting largely the low level of employment and access to schooling for persons with disability.

**Fig 1:**
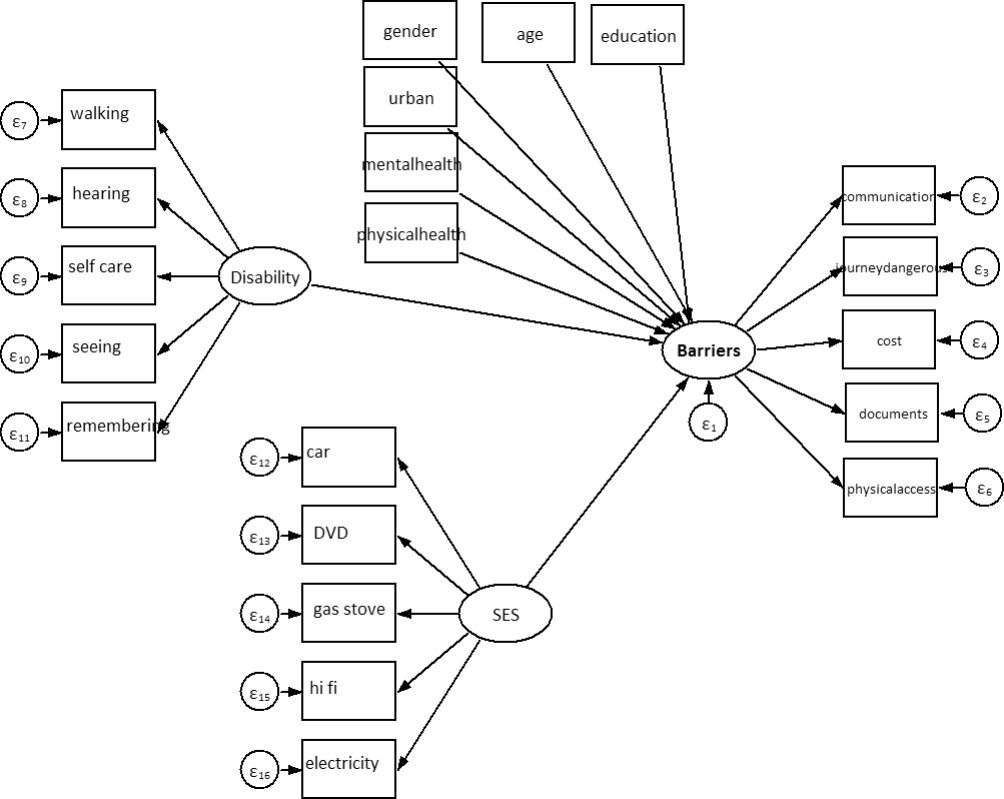
Structural equation model, simplified.

The initial part of the analysis consist of descriptive statistics and bivariate regressions. Based on these results, we subsequently developed a structural equation model (SEM) [28], which served to estimate the relationship between environmental barriers, disability, health, sociodemographic variables such as gender, age (years), education (highest level completed), urban or rural location, and socio-economic status (SES) measured through an asset scale (see models in Fig 1). The SES scale measures the number of possessions (i.e. electricity, beds, refrigerator, motorcycle, bicycle) in the household. Items were selected based on an initial principal component factor analyses. Of the inital 27 items, 9 items were excluded due to low factor loadings (< .30). The remaining items all had loadings between .35 - .86 (factor Eigenvalue 7.49, Bartlett's KOM of 0.95. Cronbach's alpha was 0.92, with an average inter-item covariance of .06). The advantage of using SEM is that it allows us to measure disability, environmental barriers and SES as latent variables. Another attractive feature of the SEM framework is that it allows for easy comparison of associations between latent and observed variables among different groups. Hence, in the final part of the analysis, we used SEM to compare the estimated relations in the four study contexts.

### Ethical considerations

As described elsewhere [24], ethical clearance was obtained from the Research and Ethical Committee, Afhad University, and The National Scientific and Research Committee, Federal Ministry of Health (Sudan), Health Research Ethics Committee, Stellenbosch University (South Africa), Office of the Permanent Secretary, Ministry of Health and Social Services (Namibia), the National Health Sciences Research Committee (Malawi), and the Norwegian Social Science Data Services. Procedures for ensuring voluntary participation, confidentiality and anonymity were included in the training and observed during data collection.

## Results

### Sample characteristics

The dataset used for the present analysis combines household and individual level data. The combined file comprises a total of 9,307 individuals. A small number of missing values, particularly in Sudan, leads to minor variations in N between different variables. Table 1 presents summary statistics for the full sample, broken down by country. The variables included in the table are described below.

**Table 1:** Sample characteristics, by country.

Variable			Total	South Africa	Namibia	Malawi	Sudan	Min	Max
Male			0∙39	0∙30	0∙41	0∙45	0∙41	0	1
Age (years)			36∙50 (20∙91)	41∙99 (18∙07)	43∙21 (23∙36)	27∙94 (18∙57)	39∙30 (21∙54)	1	100
Urban			0∙28	0∙47	0∙48	0∙02	0∙35	0	1
Education									
	No formal education		0∙15	0∙17	0∙24	0∙12	0∙05	0	1
	Less than primary school		0∙13	0∙18	0∙27	0∙06	0∙07	0	1
	Completed primary school		0∙45	0∙32	0∙31	0∙65	0∙40	0	1
	Secondary school		0∙14	0∙19	0∙12	0∙11	0∙11	0	1
	Tertiary school		0∙02	0∙04	0∙04	0∙04	0∙004	0	1
Asset scale			0∙23 (0∙26)	0∙40 (0∙29)	0∙31 (0∙27)	0∙05 (0∙08)	0∙22 (0∙16)	0	1
Activity limitation scale			1∙23 (0∙37)	1∙20 (0∙33)	1∙33 (0∙41)	1∙13 (0∙22)	1∙48 (0∙56)	1	4
Environmental barrier scale			1∙51 (0∙66)	1∙55 ((0∙71)	1∙77 (0∙79)	1∙37 (0∙48)	1∙49 (0∙81)	1	5
*N*		* *	*9∙307*	*2∙824*	*1∙624*	*1∙526*	*1∙333*	* *	* *

Mean values with standard deviation in brackets (continuous variables only). For dichotomous variables, the mean value represents the share of sample which takes the value of 1.

With the exception of the variable Urban in the Namibian sample, all country level differences are statistically significant on a .01 level or lower.

The gender balance varied significantly between the four country samples. Some of these differences, and the particularly skewed gender balance in SA, are assumed to be due to characteristics of the selected sites, with a high proportion of migrating workforce. Age did also vary significantly between the countries, and between men and women. Mean age for men and women in the total sample was 34∙7 and 37∙9 years respectively, and the gender difference in age varied somewhat between the four countries from 3∙8 years higher mean age among females in South Africa, 1∙5 years higher among females in Malawi, and 1 year higher among males in Namibia and Sudan.

### Environmental barriers

Table 2 shows the distribution of the CHIEF items for persons with (PWD) and without disabilities (Control).

**Table 2:** Frequency of experiencing barriers, in percent.

Environmental barrier	Never		Less than monthly		Monthly		Weekly		Daily	
(CHIEF items)	PWD	Control	PWD	Control	PWD	Control	PWD	Control	PWD	Control
Transportation	55.4	75.1	11	8.5	13.9	7	7.4	2.8	12.3	6.5
Natural environment	62.1	81.1	13.6	8.8	11.4	4.6	6.3	2.7	6.7	2.9
Surroundings	70.8	84.3	9.6	5.6	6.7	3	6.3	3.9	6.7	3.2
Information	74.4	85.2	8.5	5.2	6	3	3.7	2	7.4	4.6
Health care services	63.5	76.4	13.6	10.9	12.6	8	4.9	2.2	5.4	2.6
Someone's help at home	68.8	79.5	9.3	7.3	7.7	5.8	5.4	3.5	8.8	3.9
People's attitudes at home	84.9	89.9	5.9	4.8	3.7	2.4	1.9	1.4	3.7	1.6
Prejudice	83.6	88.5	5.4	4.9	3.9	3.4	3.1	1.4	3.9	1.8
N									6,909–9,009	

Overall, Table 2 indicates that persons without disabilities (i.e. those scoring no difficulty in all of the activities on the WG Short Set) have less often experienced barriers than persons with disabilities. For all items, there is a statistically significant difference between persons with and without disabilities. Between 13–45% of persons with disabilities in our sample have experienced at least one of the items listed in the CHIEF questionnaire, while the corresponding figures for persons without disabilities varies between 10–25%. The difference between persons with and without disabilities is largest for the items transportation, the natural environment and access to health care services; while the smallest differences was observed for the items attitudes at school and home and prejudice. Similarly, there are significant differences at the country level regarding the frequency of the barriers. A question assessing perception of the *extent* of the barriers revealed that the three largest barriers were transport, the natural environment and access to health care (ranked number one, two and three, respectively).

An exploratory factor analysis was conducted to assess the appropriateness of constructing an Environmental Barriers Scale of the eight items in CHIEF (Table 2). This yielded strong support for a one component solution, with an Eigenvalue of 2.45, factor loadings ranging from .37-.65, and Bartlett's KMO of 0.82. Cronbach's alpha was 0.78, with an average inter-item covariance of .036, implying strong support for a scale. The scale was constructed by adding the eight items together, dividing by number of items and finally reversed. This yields a range of 1 to 5, where 1 corresponds to never having experienced any of the barriers, and 5 to experiencing all of them daily. The mean value was 1.51, with a standard deviation of 0.66. Mean was 1.66 for persons with disabilities and 1.36 for persons without disabilities (F = 466.89, p < .001). Differences in mean value on the scale varied somewhat between the countries (F = 3444.66, p < .001), with Namibia scoring highest (1.77), followed by South Africa (1.55), Sudan (1.49), and Malawi (1.37). In all the country samples, persons with disabilities scored significantly higher than persons without disabilities.

### Bivariate regressions

In order to develop a model explaining the relationship between activity limitations (disabled individuals) and environmental barriers, bivariate linear regressions were carried out (Table 3). The analysis was done separately for persons with (PWD) and without disabilities (controls).

**Table 3:** Bivariate regressions on environmental barriers, persons with and without disabilities.

	Case			Control		
		95% CI			95% CI	
Variables	B	LB	UB	B	LB	UB
Gender (male = 1, female = 0)	-0.024	-0.07	0.026	-0.052**	-0.08	-0.02
Age (years)	0.005**	0.004	0.006	0.003**	0.002	0.004
Urban/rural (urban = 1)	-0.092**	-0.15	-0.04	-0.027	-0.06	0.008
Education	-0.141**	-0.17	-0.12	-0.047**	-0.06	-0.03
Disability (AL)	0.507**	0.442	0.572	0.788*	0.179	1.398
SES	-0.252**	-0.355	-0.150	-0.012	-0.046	0.070
Self-reported Physical health (1 = poor, 5 = very good)	-0.202**	-0.23	-0.17	-0.079**	-0.1	-0.06
Self-reported Mental health (1 = poor, 5 = very good)	-0.203**	-0.23	-0.17	-0.106**	-0.13	-0.08

* p<0.05

** p<0.01. B coefficients and 95 percent confidence intervals (lower bounds (LB) and upper bounds (UB))

According to the bivariate regression analyses, perceived environmental barriers are higher among rural respondents, increase with age and severity of disability, and lower for those with a higher level of education and with better physical and mental health. Gender has an impact only among persons without disabilities, where women report more barriers than men. Higher socioeconomic status, on the other hand, is associated with more barriers only among people with disabilities.

### A structural equation model (SEM) of environmental barriers

Based on the bivariate regressions reported above, we included all the variables from Table 3 in the subsequent SEM analysis in a combined model (Fig 1) for persons with and without disabilities. Items for the latent variables Possessions and Environmental barriers were included based on the exploratory factor analysis as described above. Attempts to drop statistically insignificant variables did not improve model fit or otherwise change the results, so all variables from the bivariate regressions were kept in the reported models.

Initially, we controlled for the different country contexts by including dichotomous variables for each country (Model 1). Subsequently, we estimated a group-wise model where estimates were allowed to vary with country, testing whether the estimates are invariant across country (Model 2). Table 4 reports the results.

**Table 4:** Structural equation model of environmental barriers (latent).

	Model 1			Model 2		
Variable	Overall	South Africa	Namibia	Malawi	Sudan	*χ*^*2*^ test^1^
Gender (male = 1, female = 0)	-0.031	-0.140	0.100	-0.018	-0.024	14.41***
	(1.70)	(3.54)**	(1.94)	(0.93)	(0.29)	
Age (number of years)	0.001	-0.001	0.001	0.001	-0.003	6.16
	(1.94)	(0.90)	(0.81)	(2.17)*	(1.35)	
Urban (urban = 1, rural = 0)	-0.077	-0.124	-0.140	0.115	0.009	12.89***
	(2.92)**	(2.78)**	(2.36)*	(1.90)	(0.10)	
Education (highest level completed)	0.005	0.012	0.019	0.035	-0.009	1.65
	(0.50)	(0.59)	(0.69)	(2.77)**	(0.21)	
SES (latent)	-0.268	-0.221	-0.298	1.309	-0.953	93.98***
	(7.09)**	(3.93)**	(3.67)**	(7.28)**	(4.12)**	
Disability (latent)	1.199	0.946	1.468	2.659	0.957	55.41***
	(13.97)**	(6.72)**	(7.12)**	(12.06)**	(6.02)**	
Physical health (1 = poor, 4 = very good)	-0.055	-0.005	-0.223	0.045	-0.049	41.67***
	(3.87)**	(0.18)	(5.85)**	(2.59)**	(0.67)	
Mental health (1 = poor, 4 = very good)	-0.090	-0.097	0.068	-0.033	-0.226	19.41***
	(5.80)**	(3.43)**	(1.74)	(1.67)	(3.15)**	
Country dummy variables						
Namibia	-0.392					
	(13.23)**					
Malawi	0.135					
	(4.88)**					
Sudan	-0.309					
	(8.26)**					
Log likelihood	-214,620.44					-147,639.04
χ^2^ model vs saturated	24,239.712***					38,300.76***
χ^2^ saturated vs baseline	117,123.04***					86,376.66***
AIC	429,492.88					295,860.09
BIC	430,367.94					297,881.07
CFI	0.798					0.574
TLI	0.78					0.569
RMSEA	0.065					0.083
SRMR	0.058	0.072	0.071	0.091	0.119	0.09
CD	0.988	0.985	0.981	0.915	0.935	0.937
N						7,669

Note: B coefficients, standard deviations in brackets.

* p<0.10

** p<0.05

*** p<0.01.

AIC: Akaike's information criterion. BIC: Bayesian information criterion. CFI: Comparative fit index. TLI: Tucker-Lewis index. RMSEA: Root mean squared error of approximation. SRMR: Standardized root mean square residual. CD: Coefficient of determination.

Starting with Model 1, Table 4 shows that activity limitation is significantly associated with environmental barriers when controlling for a number of other individual characteristics, thus confirming the initial analyses (Table 3). Also socioeconomic status, measured through a latent asset scale, is significantly and strongly associated with environmental barriers, but negatively so: the more possessions you have the fewer barriers you experience/perceive. It is worth noting that some of the associations identified in the initial bivariate regressions do not hold in the more complex model. Hence, age, gender and education are only indirectly associated with higher barriers; controlling for health and SES, these characteristics are not significantly associated with experiencing more barriers. Of the sociodemographic characteristics, only SES and rural location are associated with environmental barriers after controlling for other variables.

Finally, the model shows that respondents in Namibia report of the highest level of barriers, followed by South Africa, Sudan and Malawi. Since the model controls for individual level characteristics in the respective populations, these differences reflect the actual frequency of experienced obstacles that exist; they are not a product of, e.g., higher levels of disability or a more unhealthy population. Hence, of the country characteristics outlined initially (see description of study context above), dispersion and long distances appear to be the most detrimental for access.

**Fig 2:**
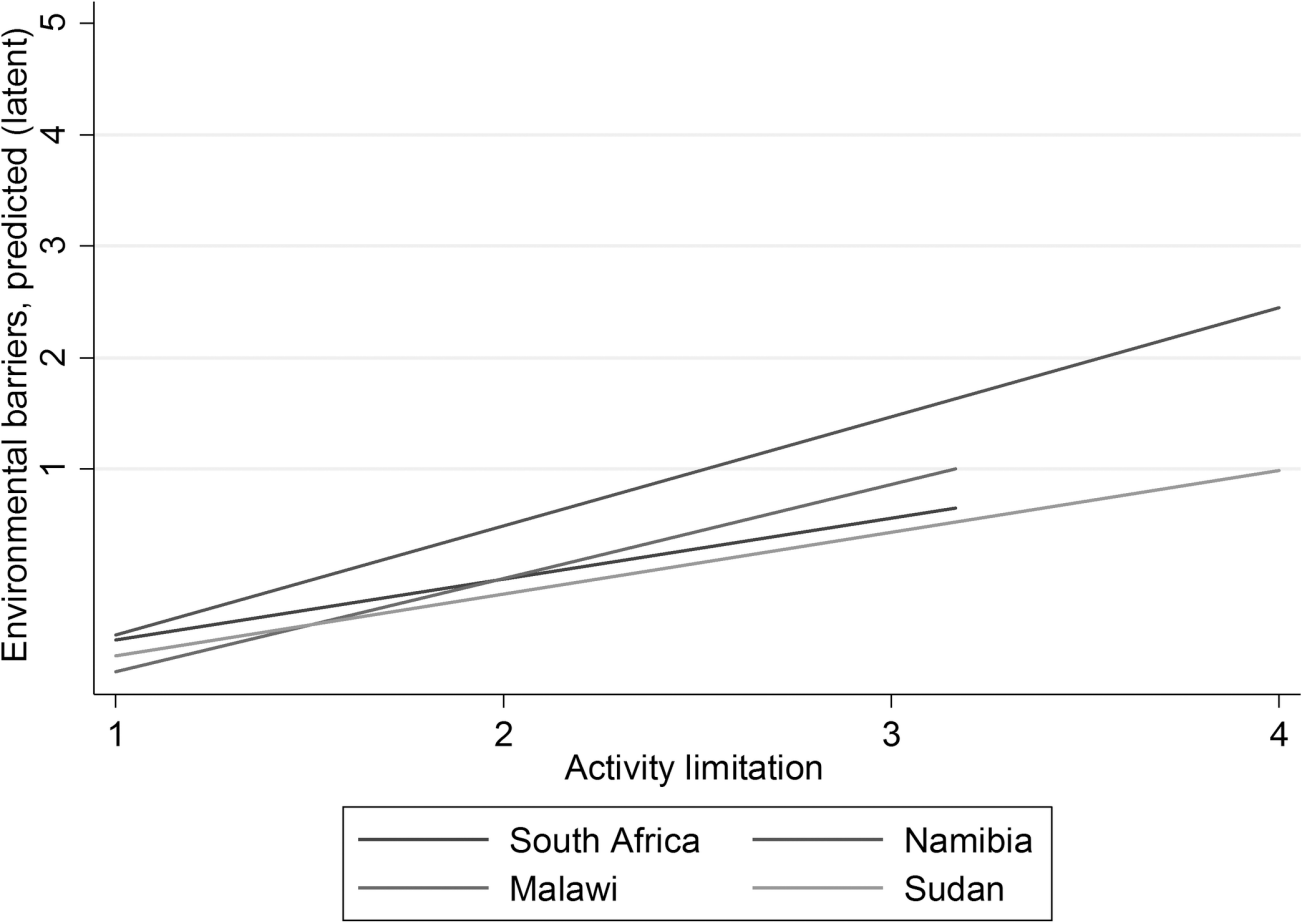
Association between activity limitations and environmental barriers.

Turning to Model 2, there appears to be several differences between countries in individual-level determinants of environmental barriers. According to Model 2, all the variables but age varies significantly with country (Χ^2^ test). Even so, some associations are fairly similar across country. Notably, activity limitation is positively associated with environmental barriers in all four settings. As illustrated in Fig 2, the association is significantly stronger in Malawi and Namibia, and weaker in South Africa and Sudan.

Surprisingly, socio-economic status appears to be strongly and positively associated with environmental barriers in Malawi. However, additional analyses (country-specific models using SEM as well as ordinary least square) show that this result is not robust to different model specifications. The mean number of possessions per inhabitant is much lower in Malawi than in the other contexts, which could potentially explain this result in the group-wise model.

The goodness of fit statistics reported at the bottom of Table 4 indicate that the models do not represent a particularly close fit to the data; however, Model 1 fares clearly better than Model 2. This was as expected, given the complex model and the large samples. Model 2 is also more complex than Model 1. Several test statistics are known to negatively bias larger samples and more complex models [29]. Closer inspection reveals that the problem is mostly due to the fact that many variables have different distributions in the four samples. Indeed, given the differences between the four populations it may simply not be possible to identify a model that fares well across countries. Most items in the SES scale are much less frequent in the Malawi sample, reflecting the widespread poverty in the country. Reducing the number of items included to measure SES improved the model fit, but did not solve the problem. However, additional analyses with alternative model specifications (i.e., persons with disabilities separately, countries separately, and using different estimation techniques (OLS regressions with additive scales instead of latent variables) confirm most of the associations reported in Table 4, with the notable exception of the effect of SES in Malawi.

## Discussion

The present study found that individuals with disabilities face more severe environmental barriers than non-disabled individuals in each of the four country subsamples. Both persons with and persons without disabilities experienced transportation, the natural environment, and accessing health care services, as being the most severe and the most frequent barriers. The rank order among the different barriers is largely the same for persons with and without disabilities. Environmental barriers were found to increase with reduced SES status and increased activity limitations. With less predictive strength, rural residence, poor mental health and physical health also predicted experiences/perceptions of greater environmental barriers. While statistically significant in the initial, bivariate analyses, education was not associated with environmental barriers in subsequent analyses, which also included disability, health and socioeconomic status. One interpretation of this is that education reduces barriers only insofar as it improves livelihood.

The association between activity limitations and environmental barriers was consistently positive across countries. The effect was strongest in Malawi and Namibia, and somewhat weaker in Sudan and South Africa. Transportation and natural environment are well documented as main barriers in the daily life of individuals in the current contexts. Transport is often challenging in Africa due to a number of reasons such as rough terrain, flooding, poor road infrastructure, huge distances; expensive, inadequate or non-existent public transport; and fewer people owning motorised vehicles [30]. The natural environment as a barrier feeds into the transportation problem and is naturally more problematic in poor contexts due to lack of infrastructure, such as poor roads, difficult terrain such as hills, rivers, extreme heat, and so on. With regards to health care services as a barrier, this may also be linked to lack of transportation and the natural environment, in combination with distances and general poverty, and limited health care provision in poorer contexts.

Overall, the study confirmed the expected association between activity limitations and environmental barriers that was also described by Reinhardt et al [12]; while also expanding these findings beyond spinal cord injury to all types of disability, as measured by the Washington Group Questions. We also found that even when controlling for other relevant variables, there is a unique association between experiences/perceptions of barriers and disability/activity limitation, thus supporting the stated hypothesis. This further confirms that specific targeted measures to accommodate persons with disability are necessary to reduce barriers and promote equitable social inclusion. Also, the role of socio-economic status (SES) as a factor influencing experiences/perceptions of barriers is as expected, confirming the important role of poverty reduction in promoting access and inclusion [31]

While this study offers unique data from sub-Saharan Africa, the four country samples are not representative at a country level, and sampling was directed by bringing forth the uniqueness of each context, with variation in rationale for selecting study sites. Any interpretation and use of results should take this into consideration. The self-report nature of the research should also be acknowledged. It should further be noted that the data collection was complex and undertaken under demanding circumstances by different teams in each country and in populations, which may have influenced the quality and introduced bias in the data. We have countered such problems through a series of workshops to ensure a joint understanding of the research process among the country teams, including active participation of the project lead as well as the work package leader in all training at country level. While the limitations still need to be acknowledged, the main aim of the article was not to present representative estimates at country level but to analyse a theoretically based model. Nonetheless, our results indicate that the experience of increased frequency and intensity of experienced barriers is strongly associated with reported activity limitations across the four country samples; albeit to different extents in different countries. This finding suggests that that this is a general mechanism that is at work in different contexts and different countries and should be a primary target for policy to address.

## Conclusion

The study has confirmed that individuals with and without disability experience the same type of environmental barriers, but that disability (operationalised in this study as having activity limitations and accounting for some important intervening variables) exacerbates the experience of these barriers. A broad approach to reduce barriers and implement measures to provide necessary support and adaptation to individuals with disability is necessary to achieve full inclusion is society among vulnerable groups. Reducing barriers for the general population would go some way to reducing the impact of these for persons with activity limitations, but additional and specific adaptations will be required to ensure full reduction of their impact and promoting equitable access, inclusion and participation in society.

## Supporting information

S1 File S_1_EquitAble_household v10 English and Chichewa Version. **Pdf.** Household questionnaire.
(PDF)

S2 File S_2_EquitAble_indivudual v10_1 English and Chichewa. **Pdf.** Individual questionnaire.
(PDF)

S3 File S_3_EquitAble_Control v10_1 English and Chichewa. **Pdf.** Control questionnaire.
(PDF)

S4 File Data used for analysis. (ZIP)

## References

[1] World Health Organization International Classification of Functioning, Disability and Health. World Health Organization: Geneva; 2001

[2] Schneidert M, Hurst R, Miller J, Ustün B. The role of the environment in the International Classification of Functioning, Disability and Health. Disability and Rehabilitation, 2003; 25(11):588–595. 12959332 10.1080/0963828031000137090

[3] Ma E, Threats TT, Worrall LE. An introduction to the International Classification of Functioning, Disability and Health for speech-language pathology: Its past, present and future. International Journal of Speech-Language Pathology, 2008;10(1–2):2–8.

[4] Vanleit B. Using the ICF to address needs of people with disabilities in international development: Cambodian case study. Disability and Rehabilitation, 2008; 30(12–13):991–998. doi: 10.1080/09638280701800251 18484394 10.1080/09638280701800251

[5] Kostanjsek N. Use of the International Classification of Functioning, Disability and Health (ICF) as a conceptual framework and common language for disability statistics and health information systems. BMC Public Health, 2011;11(Suppl 4):S3. 10.1186/1471-2458-11-S4-S3 PMC3104216 21624189

[6] Howe TJ. The ICF contextual factors related to speech-language pathology. International Journal of Speech-Language Pathology, 2008;10(1–2):27–37.

[7] Hammel J, Magasi S, Heinemann A, Gray DB, Stark S, et al Environmental barriers and supports to everyday participation: A qualitative insider perspective from people with disabilities. Archives of Physical Medicine and Rehabilitation, 2015; 96(4):578–88. doi: 10.1016/j.apmr.2014.12.008 25813890 10.1016/j.apmr.2014.12.008

[8] Wang PP, Badley EM, Gignac MP. Exploring the role of contextual factors in disability models. Disability and Rehabilitation, 2006; 28(2):135–140. doi: 10.1080/09638280500167761 16393844 10.1080/09638280500167761

[9] Visser M, van den Berg-Emons R, Sluis T, Bergen M, Stam H. Barriers to and facilitators of everyday physical activity in persons with a spinal cord injury after discharge from the rehabilitation centre. J Rehabil Med, 2008;40:461–467. doi: 10.2340/16501977-0191 18509562 10.2340/16501977-0191

[10] Algure'n B, Lundgren–Nilsson ASA, Sunnerhagen KS. Facilitators and barriers of stroke survivors in the early post-stroke phase. Disability and Rehabilitation, 2009;31(19):1584–1591. doi: 10.1080/09638280802639004 19479517 10.1080/09638280802639004

[11] Cerniauskaite M, Quintasie R, Boldt C, Raggi A, Cieza A, Bickenbach JE et al Systematic literature review on ICF from 2001 to 2009: Its use, implementation and operationalisation. Disability and Rehabilitation, 2011; 33(4): 281–309. doi: 10.3109/09638288.2010.529235 21073361 10.3109/09638288.2010.529235

[12] Reinhardt JD, Mansmann U, Fellinghauer BAG, Strobl R, Grill E, von Elm E et al Functioning and disability in people living with spinal cord injury in high- and low-resourced countries: A comparative analysis of 14 countries. Int J Public Health, 2011; 56:341–352. doi: 10.1007/s00038-010-0222-8 21165668 10.1007/s00038-010-0222-8

[13] Eide AH, Jelsma J, Loeb M, Maart S, KaToni M. Exploring ICF components in a survey among Xhosa speakers in Eastern & Western Cape, South Africa. Disability and Rehabilitation, 2008;30(11):819–829. doi: 10.1080/09638280701390998 17852254 10.1080/09638280701390998

[14] O’Donnovan M, Doyle A, Gallahar P. Barriers, activities and participation: Incorporating ICF into service planning datasets. Disability and Rehabilitation, 2009; 31(25):2073–2080. doi: 10.3109/09638280902918738 19888837 10.3109/09638280902918738

[15] Cawood J, Visagie S. Environmental factors influencing participation of stroke survivors in a Western Cape setting. African Journal of Disability, 2015;4(1):Art. #198 10.4102/ajod.v4i1.198 PMC5433486 28730037

[16] Maart S, Eide A, Jelsma J, KaToni M. Environmental barriers experienced by urban and rural disabled people in South Africa. Disability & Society, 2007;22(4):357–369.

[17] World Health Organization. World Report on Disability. World Health Organization: Geneva; 2011.

[18] Randström KB, Asplund K, Svedlund M. Impact of environmental factors in home rehabilitation − a qualitative study from the perspective of older persons using the International Classification of Functioning, Disability and Health to describe facilitators and barriers. Disability & Rehabilitation, 2012; 34(9):779–787. 22004413 10.3109/09638288.2011.619621

[19] Whiteneck G, Meade MA, Dijkers M, Tate DG, Bushnik T, Forchheimer MB. Environmental factors and their role in participation and life satisfaction after spinal cord injury. Arch Phys Med Rehabil, 2004;85(11):1793–1803. 15520974 10.1016/j.apmr.2004.04.024

[20] Carpenter C, Forwell SJ, Jongbloed LE, Backman CL. Community participation after spinal cord injury. Arch Phys Med Rehabil, 2007;88 (4), 427–433. doi: 10.1016/j.apmr.2006.12.043 17398242 10.1016/j.apmr.2006.12.043

[21] Munsaka E, Charnley H. We do not have chiefs who are disabled: Disability, development and culture in a continuing complex emergency. Disability & Society, 2013; 28(6):756–769.

[22] Amin M, MacLachlan M, Mannan H, El Tayeb S, El Khatim A, Swartz L et al EquiFrame: A framework for analysis of the inclusion of human rights and vulnerable groups in health policies. Health & Human Rights, 2011;13(2):1–20. 22957368

[23] MacLachlan M, Amin M, Mannan H, El Tayeb S, Bedri N, Swartz L et al Inclusion and human rights in African health policies: Using EquiFrame for comparative and benchmarking analysis of 51 policies from Malawi, Sudan, South Africa & Namibia. PLoS One, 2012;7(5):e35864 doi: 10.1371/journal.pone.0035864 22649488 10.1371/journal.pone.0035864 PMC3359320

[24] Eide A H, Mannan H, Khogali M, van Rooy G, Swartz L, Munthali A et al Perceived barriers for accessing health services among individuals with disability in sub-Saharan Africa. Plos One, 2015;10(5): e0125915 doi: 10.1371/journal.pone.0125915 25993307 10.1371/journal.pone.0125915 PMC4489521

[25] Washington Group on Disability Statistics. Rationale for the Short Set. 2010. Retrieved from http://www.cdc.gov/nchs/washington_group/wg_rationale.htm.

[26] Eide A H, Jele B. Living Conditions among people with disabilities in Swaziland. A national representative study. SINTEF A 20047. SINTEF Technology & Society: Oslo; 2011.

[27] Whiteneck GG, Harrison-Felix CL. Quantifying environmental factors: a measure of physical, attitudinal, service, productivity, and policy barriers. Archives of physical medicine and rehabilitation, 2004;85(8):1324–1335. 15295760 10.1016/j.apmr.2003.09.027

[28] Kline RB. Principles and Practice of Structural Equation Modeling. 4th ed. New York: Guilford Press; 20016.

[29] Hooper D, Coughlan J, Mullen M. Structural Equation Modelling: Guidelines for Determining Model Fit. Electronic Journal of Business Research Methods, 2008;6(1): 53–60.

[30] Porter G. Transport services and their impact on poverty and growth in rural Sub-Saharan Africa: A review of recent research and future research needs. Transport Reviews: A Transnational Transdisciplinary Journal, 2014;34(1):25–45.

[31] Mitra S, Posarac A, Vick B (2011) Disability and Poverty in Developing Countries: A Snapshot from the World Health Survey. SP Discussion Paper No. 1109. Washington DC: The World Bank.

