# Supplementary material for: Factors related to environmental barriers experienced by persons with and without disabilities in diverse African settings
Source: PLoS One. 2017 Oct 12;12(10):e0186342. doi: 10.1371/journal.pone.0186342 (PMC5638520; doi:10.1371/journal.pone.0186342)
Supplement: S1 File — Pdf. Household questionnaire. (PDF) [file pone.0186342.s001.pdf]

IDENTIFICATION OF HOUSEHOLD	CODE
NAME AND CODE OF REGION* ____________________	☐☐
NAME AND CODE OF DISTRICT ____________________	
NAME AND CODE OF TA* ____________________	☐☐
NAME AND CODE OF HF CATCHMENT AREA * ____________________	☐☐
ENUMERATION AREA NUMBER ……………………	☐☐☐☐☐☐☐☐☐
LOCATION 1 = urban 2 = rural ……………………	☐
HOUSEHOLD NUMBER/ID ……………………	☐☐
NAME OF HOUSEHOLD HEAD ____________________	
TOTAL NUMBER OF PERSONS IN HOUSEHOLD (the same as last Line Number filled in Section A)	☐☐
TOTAL NUMBER OF ELIGIBLE PERSONS FOR INDIVIDUAL INTERVIEW	☐☐
LINE NO. OF PRIMARY RESPONDENT	☐☐

TO BE COMPLETED BY THE INTERVIEWER	DATE OF INTERVIEW
Time interview starts ☐☐ ☐☐ Time completed ☐☐ ☐☐	Day ☐☐
Name of interviewer: ____________________ ☐☐	Month ☐☐
Comments:	Year 2 0 1 1
Signature ____________________	

SUPERVISOR	INTERVIEW STATUS	Enumerator has to return to the household	CHECKED by the Supervisor
Name : ____________________ ☐☐	Complete ☐ Incomplete ☐	Yes ☐ No ☐	☐
Signature ____________________			

GPS INFORMATION

Accuracy ☐☐ Feet

Latitude ☐ N/S Degrees ☐☐ ⁰ Minutes ☐☐ . ☐☐☐ ,

Longitude ☐ E/W Degrees ☐☐ ⁰ Minutes ☐☐ . ☐☐☐ ,

***Codes for REGION, CONSTITUENCY AND LOCALITY see separate sheet**

SECTION A: HOUSEHOLD COMPOSITION: FOR ALL PERSONS

LINE NO.	WHO ARE PERMANENT MEMBERS OF THIS HOUSEHOLD?	RELATIONSHIP TO HEAD OF HOUSEHOLD	SEX		AGE	MARITAL STATUS	ILLNESS/INJURY
	List the first names and first letter of the surname of all persons in this household, starting with the head of the household	What is the relationship of (NAME) to the head of the household? * *Kodi pali ubale wanji pakati pa (Dzina) ndi inuyo/ mutu wa banja lino?*	Is (NAME) male or female? 1=Male 2=Female		How old was (NAME) at his/her last birthday? *Kodi (Dzina) ali ndi zaka zingati?* **Enter age in completed years** 98=Don't know	What is (NAME'S) marital status?** *Kodi (Dzina) ali pa banja?* **Only 12 yrs and above**	Has (NAME) been seriously ill or injured during the past 6 months? If YES, what was the main illness or injury? *** *Kodi (Dzina) akhala akudwala kwambiri kapena kuvulala pa myezi isanu ndi umodzi yapitayi?* ***Ngati amadwala vuto lenileni linali chani****?* **Enter "00" if NO**
(1)	(2)	(3)	(4)		(5)	(6)	(7)
			M	F	IN YEARS		
01		0 1	1	2			
02			1	2			
03			1	2			
04			1	2			
05			1	2			
06			1	2			
07			1	2			
08			1	2			
09			1	2			
10			1	2			

*CODES FOR Q.3 RELATIONSHIP TO HOUSEHOLD HEAD	**CODES FOR Q.6 MARITAL STATUS	***CODES FOR Q.7 MAIN ILLNESS or injury	
01 = Head<br>02 = Spouse/living in partner<br>03 = Son/Daughter (also step-)<br>04 = Son/Daughter-in-law<br>05 = Grandchild of head/spouse<br>06 = Parent/Parent-in-law<br>07 = Brother/Sister of head/spouse<br>08 = Co-wife<br>09 = Other relatives<br>10 = Domestic worker/<br>11 = Other non-relatives<br>98 = Don't know	1 = Never married (and not cohabiting)<br>2 = Currently married<br>3 = Consensual union/Cohabiting<br>4 = Divorced/separated<br>5 = Widowed<br>8 = Don't know/refuse	01 = Cancer<br>02 = Malaria<br>03 = TB<br>04 = Bronchitis<br>05 = Cholera<br>06 = Severe diarrhoea<br>07 = Measles<br>08 = Pneumonia<br>09 = Heart disease<br>10 = High blood pressure<br>11 = Diabetes<br>12 = HIV/AIDS	13 = Malnutrition<br>14 = Mental illness<br>15 = High fever/meningitis<br>16 = Epilepsy<br>17 = Physical injury<br>18 = Asthma<br>19 = Other disease or injury (specify :)<br>____________________<br>98 = Don't know

SECTION A: HOUSEHOLD COMPOSITION: FOR ALL PERSONS – cont. for household member 11 -20

LINE NO.	WHO ARE PERMANENT MEMBERS OF THIS HOUSEHOLD?	RELATIONSHIP TO HEAD OF HOUSEHOLD	SEX		AGE	MARITAL STATUS	ILLNESS/ INJURY
	List the first names and first letter of the surname of all persons in this household, starting with the head of the household.	What is the relationship of (NAME) to the head of the household? * *Kodi pali ubale wanji pakati pa (Dzina) ndi inuyo/ mutu wa banja lino?*	Is (NAME) male or female? 1=Male 2=Female		How old was (NAME) at his/her last birthday? *Kodi (Dzina) ali ndi zaka zingati?* **Enter age in completed years** 98=Don't know	What is (NAME'S) marital status?** *Kodi (Dzina) ali pa banja?* **Only 12 yrs and above**	Has (NAME) been seriously ill or injured during the past 6 months? If YES, what was the main illness or injury? *** *Kodi (Dzina) akhala akudwala kwambiri kapena kuvulala pa myezi isanu ndi umodzi yapitayi?* ***Ngati amadwala vuto lenileni linali chani****?* **Enter "00" if NO**
(1)	(2)	(3)	(4)		(5)	(6)	(7)
			M	F	IN YEARS		
11		☐☐	1	2	☐☐	☐	☐☐
12		☐☐	1	2	☐☐	☐	☐☐
13		☐☐	1	2	☐☐	☐	☐☐
14		☐☐	1	2	☐☐	☐	☐☐
15		☐☐	1	2	☐☐	☐	☐☐
16		☐☐	1	2	☐☐	☐	☐☐
17		☐☐	1	2	☐☐	☐	☐☐
18		☐☐	1	2	☐☐	☐	☐☐
19		☐☐	1	2	☐☐	☐	☐☐
20		☐☐	1	2	☐☐	☐	☐☐

IF THERE ARE MORE THAN 20 PERSONS IN THE HOUSEHOLD, PLEASE USE A CONTINUATION SHEET AND TICK THE FOLLOWING BOX ☐

SECTION B: DISABILITY SCREENING: FOR ALL PERSONS

LINE NO.	Because of a HEALTH PROBLEM… *Chifukwa cha matenda ….*										
	Does (NAME) have difficulty seeing, even if wearing glasses? *Kodi (Dzina) ali ndi vuto lili lonse lowona ngakhale atavala magalasi?*	Does (NAME) have difficulty hearing, even if using a hearing aid? *Kodi (Dzina) ali ndi vuto la kumva ngakhale akugwiritsa ntchito zipangizo zomuthandizira kumva?*	Does (NAME) have difficulty walking or climbing steps? *Kodi (Dzina) ali ndi vuto loyenda kapena kukwera masitepe ?*	Does (NAME) have any difficulty remembering or concentrating? *Kodi (Dzina) ali ndi vuto lilonse lokhuza kloyiwala kapena kukhazikika m'maganizo?*	Does (NAME) have difficulty with self-care such as washing all over or dressing? *Kodi (dzina) ali ndi vuto lili lonse lozisamalira yekha posamba ndi kuvala?*	Using the usual language, does (NAME) have difficulty communicating for example understanding or being understood? *Pogwiritsa ntchito chinenero chake, Kodi (Dzina) ali ndi vuto lilonse akafuna kulumikizana payekha?*	Does (NAME) have a problem with nerves, sadness of depression? *Kodi (Dzina) ali ndi vuto la kukhumudwa khumudwa.*	Does (NAME) have problem performing tasks that are expected of people of their age? *Kodi (Dzina) ali ndi vuto kugwira ntchito zimene anthu amusinthu wake amayenera kugwira?*	Mark **X** person w. an activity limitation	Is (NAME) **5 yrs old** or above?<br>YES → Q.15<br>NO → STOP<br>**CHECK Q.5**	
	1: NO<br>2: SOME<br>3: A LOT<br>4: UNABLE	1: NO<br>2: SOME<br>3: A LOT<br>4: UNABLE	1: NO<br>2: SOME<br>3: A LOT<br>4: UNABLE	1: NO<br>2: SOME<br>3: A LOT<br>4: UNABLE	1: NO<br>2: SOME<br>3: A LOT<br>4: UNABLE	1: NO<br>2: SOME<br>3: A LOT<br>4: UNABLE	1: NO<br>2: SOME<br>3: A LOT<br>4: UNABLE	1: NO<br>2: SOME<br>3: A LOT<br>4: UNABLE			
**(1)**	**(8)**	**(9)**	**(10)**	**(11)**	**(12)**	**(13)**	**(14)**	**(15)**	**(16A)**	**(16B)**	
										YES	NO
01										1	2
02										1	2
03										1	2
04										1	2
05										1	2
06										1	2
07										1	2
08										1	2
09										1	2

SECTION B: DISABILITY SCREENING: FOR ALL PERSONS

LINE NO.	Because of a HEALTH PROBLEM… *Chifukwa cha matenda ….*										
	Does (NAME) have difficulty seeing, even if wearing glasses? *Kodi (Dzina) ali ndi vuto lili lonse lowona ngakhale atavala magalasi?*	Does (NAME) have difficulty hearing, even if using a hearing aid? *Kodi (Dzina) ali ndi vuto la kumva ngakhale akugwiritsa ntchito zipangizo zomuthandizira kumva?*	Does (NAME) have difficulty walking or climbing steps? *Kodi (Dzina) ali ndi vuto loyenda kapena kukwera masitepe ?*	Does (NAME) have any difficulty remembering or concentrating? *Kodi (Dzina) ali ndi vuto lilonse lokhuza kloyiwala kapena kukhazikika m'maganizo?*	Does (NAME) have difficulty with self-care such as washing all over or dressing? *Kodi (dzina) ali ndi vuto lili lonse lozisamalira yekha posamba ndi kuvala?*	Using the usual language, does (NAME) have difficulty communicating for example understanding or being understood? *Pogwiritsa ntchito chinenero chake, Kodi (Dzina) ali ndi vuto lilonse akafuna kulumikizana payekha?*	Does (NAME) have a problem with nerves, sadness of depression? *Kodi (Dzina) ali ndi vuto la kukhumudwa khumudwa.*	Does (NAME) have problem performing tasks that are expected of people of their age? *Kodi (Dzina) ali ndi vuto kugwira ntchito zimene anthu amusinthu wake amayenera kugwira?*	Mark **X** person w. an activity limitation	Is (NAME) **5 yrs old** or above?<br>YES → Q.15<br>NO → STOP<br>**CHECK Q.5**	
	1: NO<br>2: SOME<br>3: A LOT<br>4: UNABLE	1: NO<br>2: SOME<br>3: A LOT<br>4: UNABLE	1: NO<br>2: SOME<br>3: A LOT<br>4: UNABLE	1: NO<br>2: SOME<br>3: A LOT<br>4: UNABLE	1: NO<br>2: SOME<br>3: A LOT<br>4: UNABLE	1: NO<br>2: SOME<br>3: A LOT<br>4: UNABLE	1: NO<br>2: SOME<br>3: A LOT<br>4: UNABLE	1: NO<br>2: SOME<br>3: A LOT<br>4: UNABLE			
**(1)**	**(8)**	**(9)**	**(10)**	**(11)**	**(12)**	**(13)**	**(14)**	**(15)**	**(16A)**	**(16B)**	
										YES	NO
11										1	2
12										1	2
13										1	2
14										1	2
15										1	2
16										1	2
17										1	2
18										1	2
19										1	2
20											

SECTION C: LEVEL OF EDUCATION AND EMPLOYMENT – AGED 5 YEARS OR ABOVE

LINE NO.	HIGHEST LEVEL COMPLETED*	LITERACY	WORK STATUS**
Transfer the **LINE NO.** of persons as listed in Sect. A who are 5 yrs old or above	What is the highest level of education [NAME] completed?*<br><br>*Kodi (Dzina) sukulu analekezera pati?*	Can (NAME) read and write in any language?<br><br>*Kodi (Dzina) angathe kuwerenga kapena kulemba mu chiyankhulo chili chonse?*<br><br>1 = YES, I CAN READ AND WRITE<br>2 = YES, I CAN READ BUT NOT WRITE<br>3 = NO<br>8 = DON'T KNOW	What is the work status of (NAME)?**<br><br>*Kodi (Dzina) amagwira ntchito yanji?*
**(1)**	**(15)**	**(16)**	**(17)**
0 1			

*CODES FOR Q.15<br>HIGHEST LEVEL OF EDUCATION COMPLETED	**CODE FOR Q.17<br>WORK STATUS
0 = No formal education<br>1 = Less than primary school<br>2 = Primary school<br>3 = Secondary school/ High school<br>(or equivalent)<br>4 = Tertiary level education<br>5 = Other, specify …………………<br>8 = Don't know	01 = Paid work<br>02 = Self employed, such as own business or farming<br>03 = Non-paid work such as volunteer or charity<br>04 = Student/learner<br>05 = Keeping house/homemaker (unpaid)<br>06 = Retired<br>07 = Unemployed (health reasons)<br>08 = Unemployed (disability reason)<br>09 = Unemployed (other reasons)<br>10 = Others<br>98 = Don't know/Refuse

SECTION C: LEVEL OF EDUCATION AND EMPLOYMENT – AGED 5 YEARS OR ABOVE – cont. 11 to 20

LINE NO.	HIGHEST GRADE COMPLETED*	LITERACY	WORK STATUS**
Transfer the **LINE NO.** of persons as listed in Sect. A who are 5 yrs old or above	What is the highest standard form or level of education [NAME] completed?*<br><br>*Kodi (Dzina) sukulu analekezera pati?*	Can (NAME) read and write in any language?<br><br>*Kodi (Dzina) angathe kuwerenga kapena kulemba mu chiyankhulo chili chonse?*<br><br>1 = YES<br>2 = NO<br>8 = DON'T KNOW	What is the work status of (NAME)?**<br><br>*Kodi (Dzina) amagwira ntchito yanji?*
**(1)**	**(15)**	**(16)**	**(17)**

*CODES FOR Q.15<br>HIGHEST GRADE COMPLETED	*CODE FOR Q.17<br>WORK STATUS
0 = No formal education<br>1 = Less than primary school<br>2 = Primary school<br>3 = Secondary school/ High school (or equivalent)<br>4 = Tertiary level education<br>5 = Other<br>8 = Don't know	01 = Paid work<br>02 = Self employed, such as own business or farming<br>03 = Non-paid work such as volunteer or charity<br>04 = Student/learner<br>05 = Keeping house/homemaker (unpaid)<br>06 = Retired<br>07 = Unemployed (health reasons)<br>08 = Unemployed (disability reason)<br>09 = Unemployed (other reasons)<br>10 = Others<br>98 = Don't know/Refuse

NOTE: The following questions should be completed by the PRIMARY RESPONDENT/HEAD OF HOUSEHOLD

SECTION D: INCOME AND EXPENSES

Question (18):

What is the PRIMARY source and SECONDARY source (if any) of income in your household?

Kodi ndi njira yiti yodalilika komanso njira zina yopezera ndalama zothandizira pa khomo panu?

**Income Category**	**Primary source** *[Circle **one** only]*	**Secondary source** *[Circle **one** only]*
a. Wage/Salary work	01	01
b. Remittances received	02	02
c. Cash cropping	03	03
d. Livestock sales	04	04
e. Subsistence farming/fishing	05	05
f. *Ganyu* (Casual labour)	06	06
f. Social cash transfer	07	07
i) Poverty cash transfer	08	08
ii) Disability grant	09	09
iii) Child support grant	10	10
iv) Old age pension	11	11
v) Foster care grant	12	12
vi)	13	13
vi) Other (specify)	14	14
g. Formal business (registered)	15	15
h. Informal business (non-registered - see below*)	16	16
i. Private insurance/pension	17	17
j. Workman's Compensation	18	18
k. Rent	19	19
l. Other (specify)	20	20
m. No income from any source	21	
n. Not stated/Refused	22	

*This includes payments received for handicrafts, knitting, sewing, repairing shoes, repairing punctures, for providing services (e.g. making thatch roofs for huts, cutting reeds etc.). Also includes income from selling e.g. charcoal, local gin, local beer etc.

Question (19):

Are the primary sources of income stable and regular?
Kodi njira zodalilika zopezera ndalama ndi zokhazikika?

Yes	No
1	2

SECTION E: OWNERSHIP

Question (20):

Do you or anyone in your household own any of the following (in working condition)?
Kodi inu kapena wina wake pakhomo pano ali ndi zinthu izi zoti zikugwira ntchito?

	Yes	No		Yes	No
a. Radio	1	2	o. Refrigerator	1	2
b. Hi-fi/music stereo	1	2	p. Microwave	1	2
c. Internet access in the home	1	2	q. Electricity	1	2
d. DVD/VHS player	1	2	r. Solar energy system	1	2
e. Cell phone/mobile	1	2	s. Electrical generator	1	2
f. Telephone in the house	1	2	t. Personal computer	1	2
g. Iron	1	2	u. Bicycle	1	2
h. Fan	1	2	v. Motorcycle/quad bike	1	2
i. Heater	1	2	w. Dishwasher	1	2
j. Air conditioner	1	2	x. Bed(s)	1	2
k. Stove with gas/electric	1	2	y. Livestock (cattle etc.)	1	2
l. Stove with paraffin	1	2	z. Washing machine	1	2
m. Sofa	1	2	aa. Satellite dish	1	2
n. Television	1	2	bb. Car	1	2

SECTION F: POPULATION DISPLACEMENT

Question (21):

Have you and your family ever been forced or obliged to leave your home or place of habitual residence?
Kodi inu ndi banja lanu munayamba mwakakamizidwa kuchoka malo amene mumakhala?

Yes	No
1	2

→ *If the answer is NO, skip to Question 23*

Question (22):

Could you tell me the main reason that you have moved from your home? *[Circle **one** only]*
Chifukwa chenicheni chimene munachokera kumene mumakhala?

Main reason	Code
Armed conflict	1
General violence	2
Violations of human rights (political persecution)	3
Natural or human-made disasters	4
Economic reasons (unemployment, services, education, health care, etc.)	5
Farm eviction	6
Other, specify________________________________	7

Question (23):

What language do you speak most often at home? *[Circle **one** only]*

Ndi chiyankhulo chanji chomwe mumayankhula pa nyumba kawirikawiri?

Language group	Code
Chichewa	1
Chiyao	2
English	3
Chitumbuka	4
Chitonga	5
Chilomwe	6
Chisena	7
Chinkhonde	8
Other, specify________________________________	10

Question (24):

Do you feel that your language group is a minority?
Kodi anthu a chilankhulo chanu ndi ochepa?

Yes	No
1	2

SECTION G: USE OF HEALTH FACILITY

Question (25):

Do members of your household generally use the …………(NAME OF FACILITY):

Kodi anthu a munyumba mwanu amagwiritsa ntchito………. (DZINA LA CHIPATALA)

Frequency	Code
Yes, always	1
Yes, sometimes	2
Use both health care facility plus others	3
No, but use other health care facilities	4
Never use any health care facilities	5

→ *If the answer is 'YES, always', end the interview.*

Question (26):

What are the main reasons why you *never* use this facility, or only use it *sometimes,* or why you use *other* facilities?

Kodi ndi zifukwa zotani zomwe simugwiritsa ntchito chipatala chimenechi kapena mumagwiritsa ntchito mwa apo ndi apo kapena mumapita kwina?

Main reasons	Code	Main reasons	Code
a. Cost	01	l. There are no services	12
b. No transport	02	m. Language barrier	13
c. Discrimination by health providers	03	n. Distance from home to clinic	14
d. Attitudes of health care providers	04	o. Physical accessibility of the facility	15
e. Had a bad incident and so don't go anymore	05	p. Not satisfied with outcomes of previous experience	16
f. The gender of health care provider	06	q. Opening times are not suitable	17
g. The type (professional category) of health care provider	07	r. Not sick enough or not sick (do not need)	18
h. Old age	08	s. Waiting times too long	19
i. Disability	09	t. Religious belief	20
j. Crime, danger	10	u. No knowledge about the health facility	21
k. Lack of time due to domestic or other responsibilities	11	v. Other, specify ………………………………	22

THANK YOU VERY MUCH FOR YOUR TIME!
