# Supplementary material for: Factors related to environmental barriers experienced by persons with and without disabilities in diverse African settings
Source: PLoS One. 2017 Oct 12;12(10):e0186342. doi: 10.1371/journal.pone.0186342 (PMC5638520; doi:10.1371/journal.pone.0186342)
Supplement: S2 File — Pdf. Individual questionnaire. (PDF) [file pone.0186342.s002.pdf]

EquitAble WP4 – Version 010

IDENTIFICATION OF INDIVIDUAL	CODE
NAME AND CODE OF REGION* ______________________	
NAME AND CODE OF DISTRICT ______________________	
NAME OF TA* ______________________	
NAME OF CODE OF HF CATCHMENT AREA* ______________________	
ENUMERATION AREA NUMBER	
LOCATION 1 = urban 2 = rural	
HOUSEHOLD NUMBER/ID	
NAME OF HOUSEHOLD HEAD ______________________	

DETAIL OF INDIVIDUAL

NAME ____

AGE

LINE NUMBER IN HOUSEHOLD LISTING

DISABILITY STATUS FROM THE HOUSEHOLD QUESTIONNAIRE Y N

IS THIS A FACE-TO-FACE INTERVIEW WITH THE PERSON?
[Do not read out. Code by observation]

1 = YES (i.e. interview directly with the person)
2 = NO (i.e. someone else is reporting on behalf of the person)
3 = BOTH (i.e. someone else is reporting or interpreting together with the person)

If NO or BOTH, who is the person reporting?

Line number of person as proxy

TO BE COMPLETED BY THE INTERVIEWER

Time interview starts

Time completed

Name of interviewer: ____
Comments:

Signature ______________________________

Date of interview

Day

Month

Year 2 0 1 1

SUPERVISOR

Name : ______________________

Signature ______________________

INTERVIEW STATUS		Enumerator has to return to the household		CHECKED by the Supervisor
Complete	Incomplete	Yes	No	

***Codes for REGION, CONSTITUENCY AND LOCALITY see separate sheet**

SECTION A: DIFFICULTIES AS A RESULT OF A HEALTH PROBLEM OR IMPAIRMENT

Question (1):

The next questions ask about difficulties you may have doing certain activities BECAUSE OF A HEALTH PROBLEM OR IMPAIRMENT: [*Circle only* ***one*** *per row]*

		No	Some	A lot	Unable
a)	Do you have difficulty seeing, even if wearing glasses?<br>*Kodi inuyo muli ndi vuto lili lonse lowona ngakhale mutavala magalasi?*	1	2	3	4
b)	Do you have difficulty hearing, even if using a hearing aid?<br>*Kodi inuyo muli ndi vuto la kumva ngakhale mutagwiritsa ntchito zipangizo zokuthandizirani kumva?*	1	2	3	4
c)	Do you have difficulty walking or climbing steps?<br>*Kodi inuyo muli ndi vuto loyenda kapena kukwera masitepe ?*	1	2	3	4
d)	Do you have difficulty remembering or concentrating?<br>*Kodi inuyo muli ndi vuto lilonse lokhuza kuyiwala kapena kukhazikika m'maganizo?*	1	2	3	4
e)	Do you have difficulty with self-care such as washing all over or dressing?<br>*Kodi inuyo muli ndi vuto lili lonse lozisamalira nokha posamba ndi kuvala?*	1	2	3	4
f)	Using your usual (customary) language, do you have difficulty communicating for example understanding or being understood?<br>*Pogwiritsa ntchito chinenero chanu, kodi muli ndi vuto lilonse mukafuna kulumikizana ndi anthu ena panokha?*	1	2	3	4
g)	Do you have a problem with nervousness, sadness or depression?<br>*Kodi inuyo muli ndi vuto la mantha, chisoni kapena kukhumudwakhumudwa.*	1	2	3	4
h)	Do you have a problem performing tasks that are expected of people of your age?<br>*Kodi inuyo muli ndi vuto kugwira ntchito zimene anthu amusinthu wake amayenera kugwira?*	1	2	3	4

Question (2):

In the last 30 days, how much difficulty did you have doing the following activities BECAUSE OF A HEALTH PROBLEM OR IMPAIRMENT? [*Circle only* ***one*** *per row]*

Pa masiku 30 apitawa kodi inu munavutika bwanji pochita zinthu izi chifukwa cha matenda, kapena kulumala:

		**None**<br>*Ayi*	**Mild**<br>*Pang'ono kwambi*	**Moderate**<br>*Pang'ono*	**Severe**<br>*Kwambiri*	**Extreme or cannot do**<br>*Nkosatheka*
a)	Standing for long periods such as 30 minutes?<br>*Kuimilira nthawi yayitali monga mphindi 30?*	1	2	3	4	5
b)	Taking care of your household responsibilities?<br>*Kugwira ntchito zapakhomo?*	1	2	3	4	5
c)	Learning a new task, for example, learning how to get to a new place?<br>*Kuphunzira kuchita chinthu chatsopano monga kupita malo a chilendo?*	1	2	3	4	5
d)	Joining in community activities (for example, festivities, religious or other activities) in the	1	2	3	4	5

same way as anyone else can?<br>*Kutengapo mbali pazochitika zamudera monga zikondwerero, mapemphero kapena zochitika zinamonga wina aliyense amapangira?*					
If all of Questions 2a – 2d scored "1" (none) skip Question 3 and go straight to Section B					

Question (3):

In the last 30 days, how much difficulty did you have doing the following activities BECAUSE OF A HEALTH PROBLEM OR IMPAIRMENT? [*Circle only* ***one*** *per row]*

Pa masiku 30 apitawa kodi inu munavutika bwanji pochita zinthu izi chifukwa cha matenda, kapena kulumala:

		None	Mild	Moderate	Severe	Extreme or cannot do
**a)**	Concentrating on doing something for ten minutes?<br>*Kuchita chinthu mwachidwi kwa mphindi khumi?*	1	2	3	4	5
**b)**	Walking a long distance such as a kilometer [or equivalent]?<br>*Kuyenda kwa mtunda wautali?*	1	2	3	4	5
**c)**	Washing your body? *Kusamba?*	1	2	3	4	5
**d)**	Getting dressed? *Kuvala?*	1	2	3	4	5
**e)**	Dealing with people you do not know?<br>*Kulumikizana ndi anthu oti simukuwadziwa.*	1	2	3	4	5
**f)**	Maintaining a friendship? *Kusunga ubwenzi?*	1	2	3	4	5
**g)**	Your day to day work? *Ntchito zanu za tsiku ndi tsiku?*	1	2	3	4	5

SECTION B: PAIN

Question (4): Do you have frequent pain?

Kodi mumamva kupweteka mu thupi pafupipafupi?

Yes	1
No	2

Question (5):

In the past 3 months how often did you have pain? *[Circle* ***one*** *only]*

Miyezi yitatu yapitayi mwamva kupweteka mthupi mowirikiza bwanji?

Never<br>*Palibe*	1	***Skip → Go to Q: 7***
Sometimes<br>*Nthawi zina*	2	
Most days<br>*Masiku ambiri*	3	
Every day<br>*Tsiku lili lonse*	4	

Question (6):
Thinking about the last time you had pain, how much pain did you have? *[Circle **one** only]*

Poganizira nthawi yimene munamvapo ululu mbuyomu, kodi ululu unali wochuluka bwanji?

A little<br>*Pang'ono*	1
A lot<br>*Kwambiri*	2
Somewhere in between a little and a lot<br>*Pakatikati*	3

SECTION C: QUESTIONS FOR INDIVIDUALS WITH LIMB AMPUTATIONS ONLY

Question (7)
Do you have a limb amputation? **[IF NO GO TO QUESTION 18]**
Kodi munadulidwapo mwendo kapena mukono?

No	1	***Skip → Go to Q: 18***
Yes	2	

Question (8):
Do you experience **residual limb (stump) pain** (pain in the remaining part of your amputated limb)?
Kodi mumamva ululu pamene munadulidwa?

Question (9):
During the last week, how many times have you experienced stump pain? ☐
Kodi sabata yathayi mwakhala mukumva kupweteka kangati pamene munadulidwapo?

Question (10):
How long, on average, did each episode of pain last? ☐ (minutes)
Mongoyerekeza, ululuwu umatenga nthawi yayitali bwanji?

Question (11):
Please indicate the level of stump pain experienced <u>during the last week</u> on the scale below: *[Circle **one** only]*

Kodi ululu umenewu unali wochuluka bwanji?

Excruciating<br>*Kuwawa koopsya*	1
Horrible<br>*Kuwawa kwambiri*	2
Distressing<br>*Kosautsa*	3
Discomforting<br>*Kusowetsa mtendere*	4
Mild<br>*Pang'ono*	5

Question (12):
How much did stump pain interfere with your normal lifestyle (e.g. work, social and family activities) during the last week? *[Circle **one** only]*

Kodi kuwawaku kunasokoneza bwanji moyo wanu mu sabata yapitayi?

A lot	1
Quite a bit	2
Moderately	3
A little bit	4
Not at all	5

Question (13):
Do you experience **phantom limb pain** (pain in the part of the limb which was amputated)?

Kodi mumamva kupweteka mu chiwalo chimene chinachotsedwa?

No	1	***Skip → Go to Q: 18***
Yes	2	

Question (14):
During the last week, how many times have you experienced phantom limb pain? [ ]

Kodi sabata yathayi, kupwetekaku munakumva kangati?

[***If zero, go to Q 18***]

Question (15):
How long, on average, did each episode of pain last? [ ] (minutes)

Kodi kupwetekaku kunatenga nthawi yayitali bwanji?

Question (16):

Please indicate the level of phantom limb pain experienced during the last week on the scale below:
Kodi kupwetekaku kunali kochuluka bwanji?
*[Circle **one** only]*

Excruciating<br>*Kuwawa koopsya*	1
Horrible<br>*Kuwawa kwambiro*	2
Distressing<br>*Kosautsa*	3
Discomforting<br>*Kusowetsa mtendere*	4
Mild<br>*Pang'ono*	5

Question (17):
How much did phantom limb pain interfere with your normal lifestyle (e.g. work, social and family activities) during the last week? *[Circle **one** only]*

Kodi kupwetekaku kunasokoneza bwanji moyo wanu watsiku ndi tsiku sabata yathayi?

A lot	1
Quite a bit	2
Moderately	3
A little bit	4
Not at all	5

SECTION D: FATIGUE

Question (18):
In the past 3 months, how often did you feel very tired or exhausted? *[Circle **one** only]*

Miyezi yitatu yapitayi, mumamva kutopa mowilikiza bwanji?

Never	1	***Skip → Go to Q: 21***
Some days	2	
Most days	3	
Every day	4	

Question (19):
Thinking about the last time you felt tired or exhausted, how long did it last? *[Circle **one** only]*

Mukaganizira nthawi yomaliza kutopa, kutopaku kunakutenga nthawi yayitali bwanji?

Some hours	1
Some days	2
Most days	3
Every day	4

Question (20):
Thinking about the last time you felt very tired or exhausted, how would you describe the level of tiredness?

Mukaganizira nthawi yomaliza yimene munamva kutopa kwambiri,kodi kutopa kumeneku mungakufotokoze kotani?

A little	1
A lot	2
Somewhere in between a little and a lot	3

SECTION E: HEALTH CONDITIONS

Question (21):
Tell me the health conditions that you have **[LIST ALL THE HEALTH CONDITIONS MENTIONED BY RESPONDENT]**.
In case of other illnesses, write at "Other"].

Kodi mungandiuzeko za mavuto a umoyo wanu?

*[Circle **one** for each row]*

	Condition/impairment	Yes	No
**a)**	Heart problems	1	2
**b)**	Tuberculosis and other lung problems	1	2
**c)**	Mental health problems	1	2
**d)**	Joint problems	1	2
**e)**	Malaria	1	2
**f)**	Diabetes / Sugar disease	1	2
**g)**	Blood pressure	1	2
**h)**	Cancer	1	2
**i)**	Albinism or disfigurement	1	2
**j)**	Intellectual disability	1	2
**k)**	Epilepsy / Fits / Seizures	1	2
**l)**	HIV / AIDS	1	2
**m)**	Measles	1	2
**n)**	Diarrhoea / cholera	1	2

	Condition/impairment	Yes	No
**o)**	Foetal alcohol syndrome	1	2
**p)**	Drug related problems	1	2
**q)**	Alcoholism, drug abuse	1	2
**r)**	Malnutrition	1	2
**s)**	Stroke	1	2
**t)**	Pregnancy problems	1	2
**u)**	Birth problems	1	2
**v)**	None	1	2
		1	2
**w)**	Other 1 ____________________	1	2
**x)**	Other 2____________________	1	2
**y)**	Other 3____________________	1	2
**z)**	Other 4____________________	1	2

SECTION F: PARTICIPATION RESTRICTION

For the following questions, think about the environment in which you live, including the use of assistive devices or personal support:
Mafunso otsatirawa tikambirana za malo amene mukukhala komanso zipangizo zozithandizira.
Question (22):
Do you have any difficulty doing the following? *[Read out the options and code using the codes below]*
Kodi muli ndi vuto lili lonse pochita zinthu izi?

	Participation restriction items	Score
**a)**	shopping (getting goods and services)<br>*Kugula katundu/zinthu*	
**b)**	preparing meals (cooking)<br>*Kuphika/kukonza chakudya*	
**c)**	doing housework (washing/cleaning)<br>*Kugwira ntchito za pakhomo (kuchapa)*	
**d)**	taking care of personal objects (mending/ repairing)<br>*Kusamala katundu wa iwe mwini*	
**e)**	taking care of others<br>*Kusamala anthu ena*	
**f)**	making friends and maintaining friendships<br>*Kupeza ndi kusunga maubwenzi*	
**g)**	interacting with persons in authority (officials, village chiefs)<br>*Kucheza ndi anthu amaudindo (ngati mafumu)*	
**h)**	interacting with strangers<br>*Kucheza ndi anthu achilendo*	
**i)**	creating and maintaining family relationships<br>*kupezandi kusunga ubale wa pabanja (kuyambitsa ndi kupitiliza chibale)*	

	Participation restriction items	Score
**j)**	making and maintaining intimate relationships<br>*Kuchita ndi kupitiliza zibwenzi*	
**k)**	going to school and studying (education)<br>*kuphunzira sukulu*	
**l)**	getting and keeping a job (work & employment)<br>*kupeza ndi kusunga ntchito*	
**m)**	handling income and payments (economic life)<br>*kutha kugwiritsa ntchito ndalama*	
**n)**	taking part in clubs/organisations (community life)<br>*Kutenga nawo mbali m'magulu ndi m'mabungwe*	
**o)**	taking part in recreation/leisure (sports/play/crafts/hobbies/arts/culture)<br>*kutenga nawo mbali m'masewera ndi m'zisangalalo*	
**p)**	taking part in religious/spiritual activities<br>*Kutenga nawo mbali m'zochitika za chipembezo.*	
**q)**	taking part in political life and citizenship<br>*Kutenga nawo mbali pa nkhani za ndale ndi kukhala nzika ya dziko*	

Coding
0 = No problem *(Palibe vuto)*
1 = Mild problem *(Vuto lapang'ono kwambiri)*
2 = Moderate problem *(Vuto la pang'ono)*
3 = Severe problem *(Vuto kwambiri)*
4 = Complete problem (unable to perform) (*Nkosatheka)*
8 = Not specified/not applicable (*Sizikugwirizana)*

SECTION G: ASSISTIVE DEVICES

Ask either direct or proxy respondents: please remember the information must be about the person with disability.

Question (23):

Do you use an assistive device? [*For examples, see 23 below*]

Kodi mumagwiritsa ntchito chipangizo chozithandizira?

Yes	1	**Go to Q: 24**
No	2	**Go to Q: 34**

Question (24):

Please specify which assistive devices you usually use *[Read out; circle* ***one*** *answer for each row].*

Tchulani zipangizo zozithandizira zimene mumagwiritsa ntchito. **[GIVE EXAMPLE TO EXPLAIN SOME OF THE ASSISTIVE DEVICES]**

Device	Device category	Examples:	Yes	No	Not applicable (don't need it)
	**Information *(uthenga)***				
**1**	• **Hearing aids *(Zithandizo zothandizira kumva)***	Hearing aids	1	2	3
**2**	• **Visual aid *Zipangizo zothandizira kuona)***	Eye glasses, magnifying glass, telescopic lenses/glasses, enlarge print, Braille	1	2	3
**3**	**Communication *(kulumikizana)***	Sign language interpreter, fax, portable writer, computer, picture boards, cards	1	2	3
**4**	**Personal mobility *(Mayendedwe)***				
	• **Walking *(Kuyenda)***	Crutches, walking sticks, white cane, guide, standing frame	1	2	3
	• **Wheeled mobility (*Njinga)***	Wheelchairs	1	2	3
	• **Orthoses and prostheses *(Miyendo kapena manja/mikono oyikira)***	Orthoses and prostheses	1	2	3
**5**	**Household items (*katundu wa mu nyumba)***	Flashing light on doorbell, amplified telephone, vibrating alarm clock	1	2	3
**6**	**Personal care & protection *(Zipangizo zozitetezera kapena kuzisamalira)***	Special fasteners, bath & shower seats, toilet seat raiser, commode chairs, safety rails, eating aids	1	2	3
**7**	**For handling products & goods *Zipangizo zogwirira ntchito)***	Gripping tongs, aids for opening containers, tools for gardening	1	2	3
**8**	**Computer assistive technology *(Zipangizo zothandiza kugwiritsa ntchito computer)***	Keyboard for the blind, screen reader, synthetic speech	1	2	3
**9**	**Other (*Zina)***	Specify:	1	2	3

Question (25):

Is the assistive device(s) mentioned above in good working condition/order?
*[If more than one device in one category, choose **most important** device - List device by **name**]*

Kodi zipangizo zimene mwatchulazi zikugwira ntchito pakadali pano?

Name of Device:	Good working condition?
a.	
b.	
c.	

CODING
1 = Yes
2 = No
8 = Don't know

Question (26):
Think about how much you used your assistive device over the past two weeks. On an average day, how many hours did you use it? *[Circle **one** only]*

Masabata awiri apitawa chipangizocho mudagwiritsa ntchito nthawi yayitali bwanji?

None	1
Less than 1 hour	2
1-4 hours	3
5-8 hours	4
More than 8 hours	5

Question (27):
Does your device help you as intended? *[Circle **one** only]*

Kodi chipangizocho chimagwira ntchito yake moyenera?

Does not help at all<br>*Sichithandiza*	1
Helps slightly<br>*Chimathandiza pang'ono*	2
Helps moderately<br>*Chimathandiza pakatikati*	3
Helpsquite a lot<br>*Chimathandiza kwambiro*	4
Helps very much<br>*Chimathandiza kwambiro*	5

Question (28):
How much difficulty do you still have even if you use the assistive device? *[Circle **one** only]*

Mukuvutika bwanji ngakhale muli ndi chipangizocho?

Very much difficulty<br>*Vuto lalikulu kwambiri*	1
Quite a lot of difficulty<br>*Vuto lalikulu pang'ono*	2
Moderate difficulty<br>*Vuto lapakatikati*	3
Slight difficulty<br>*Vuto pang'ono*	4
No difficulty<br>*Palibe vuto*	5

Question (29):
Has obtaining and using the device been worth the trouble? *[Circle **one** only]*
Kodi kupeza ndi kugwiritsa ntchito chipangizocho kwathandiza?

Not at all worth it<br>*Nkosapindula*	1
Slightly worth it<br>*Nkopindula pang'ono*	2
Moderately worth it<br>*Nkopindula pakatikati*	3
Quite a lot worth it<br>*Nkombindula kwambiri*	4
Very much worth it<br>*Nkopindula koposa*	5

Question (30):
Considering everything, how much has your use of assistive devices improved your quality of life? *[Circle **one** only]*
Kodi chipangizocho chasintha bwanji moyo wanu wa tsiku ndi tsiku?

Worse<br>*Kwawonjezera vuto*	1
No change<br>*Palibe chasintha*	2
Slightly better<br>*Kwasintha pang'ono*	3
Quite a lot better<br>*Kwasintha kwambiri*	4
Much better<br>*Kwasintha koposa*	5

Question (31):
Where did you get the assistive device(s)? *[Read out; record only **one** answer for each line]*
Kodi chipangizochi munachipeza kuti?

*[If more than one device in one category, choose **most important** device - List device by **name**]*

Name of Device:	Where did you get the device?*	Can you give an estimate of the cost of the device?
a.		
b.		
c.		

*CODING
1 = Private hospital
2 = Government health service
3 = Other government service (not health)
4 = NGO
5 = Other
8 = Don't know

Question (32):
Were you given any information or help on how to use your device(s)? *[Record only **one** answer for each line]*

Kodi mudapatsidwa malangizo ena aliwonse okuthandizani pa zakagwiritsidwe ntchito ka chipangizochi?

Name of Device:	Information or help
a.	
b.	
c.	

CODING
1 = Complete/full information
2 = Some information
3 = No information
8 = Don't know/ Can't remember

Question (33): Who, if any, maintains or repairs your assistive device(s)? *[Do not read out: record only **one** answer for each line]*
Kodi ndi ndani amene amakuthandizani kusamala kapena kukonza chipangizochi chikawonongeka?

*[If more than one device in one category, choose **most important** device - List device by **name**]*

Name of Device:	Maintenance /Repair
a.	
b.	
c.	

CODING
1 = Self
2 = Government
3 = Family
4 = Employer
5 = NGO
6 = Other (specify)
7 = Not maintained
8 = Cannot afford to maintain or repair it
98 = Don't know

SECTION G: INVENTORY OF ENVIRONMENTAL FACTORS

Question (34):
First, please tell me how often each of the following has been a barrier to your own participation in the activities that matter to you. Think about the past year, and tell me whether each item on the list below has been a problem **daily, weekly, monthly, less than monthly, or never.**
[Please CIRCLE only one]

Tsopano tikambirana ziphinjo zomwe zimakupangitsani kuti musatengepo mbali pa zochitika zomwe zili zofunikira kwa inu.

	1. Daily	2. Weekly	3. Monthly	4. Less than monthly	5. Never	8. Not applicable	2. Big problem	1.Little problem
**a)** In the past 12 months, how often has the availability/accessibility of transportation been a problem for you? *Kodi pamiyezi khumi ndi iwiri yapitayi, mwakumana ndi vuto la mayendedwe motani?*	1	2	3	4	5	8		
**a1)** When this problem occurs has it been a big problem or a little problem? *Vuto limeneli likapezeka limakhala lalikulu kapena laling'ono?*							1	2
**b)** In the past 12 months, how often has the natural environment – temperature, terrain, climate – made it difficult to do what you want or need to do? *Kodi pa miyezi khumi ndi iwiri yapitayi, zinthu za chilengedwe monga mapiri, zigwa ndi kusintha kwa nyengo zinakulepheletsani mowirikiza bwanji kupanga zinthu zimene mumafuna?*	1	2	3	4	5	8		
**b1)** When this problem occurs has it been a big problem or a little problem? *Vuto limeneli likapezeka limakhala lalikulu kapena laling'ono?*							1	2
**c)** In the past 12 months, how often have other aspects of your surroundings – lighting, noise, crowds, etc – made it difficult to do what you want or need to do? *Kodi pa miyezi khumi ndi iwiri yapitayi, zinthu zina zokuzungulirani monga kuwala, phokoso, kuchuluka kwa anthu, zinakulepheletsani mowirikiza bwanji kupanga zinthu zimene mumafuna?*	1	2	3	4	5	8		
**c1)** When this problem occurs has it been a big problem or a little problem? *Vuto limeneli likapezeka limakhala lalikulu kapena laling'ono?*							1	2

Question	1. Daily	2. Weekly	3. Monthly	4. Less than monthly	5. Never	8. Not applicable	2. Big problem	1.Little problem
**d)** In the past 12 months, how often has the information you wanted or needed not been available in a format you can use or understand? *Pamiyezi khumi ndi iwiri yapitayi ndi mowirikiza bwanji mwakumana ndi vuto lokanika kupeza uthenga mudongosolo lomwe inu mukhoza kugwiritsa ntchito kapena kumva?*	1	2	3	4	5	8		
**d1)** When this problem occurs has it been a big problem or a little problem? *Vuto limeneli likapezeka limakhala lalikulu kapena laling'ono?*							1	2
**e)** In the past 12 months, how often has the availability of health care services and medical care been a problem for you? *Pamiyezi khumi ndi iwiri yapitayi mwapezana ndi vuto la kapezegwe ka chisamaliro cha zaumoyo ndi mankhwala mowirikiza bwanji?*	1	2	3	4	5	8		
**e1)** When this problem occurs has it been a big problem or a little problem? *Vuto limeneli likapezeka limakhala lalikulu kapena laling'ono?*							1	2
**f)** In the past 12 months, how often did you need someone else's help in your home and could not get it easily? *Pa miyezi khumi ndi iwiri yapitayi ndi mowirikiza bwanji munafuna chithandizo pakhomo pano koma sichinapezeke?*	1	2	3	4	5	8		
**f1)** When this problem occurs has it been a big problem or a little problem? *Vuto limeneli likapezeka limakhala lalikulu kapena laling'ono?*							1	2
**g)** In the past 12 months, how often did you need someone else's help at school or work and could not get it easily? *Pa miyezi khumi ndi iwiri yapitayi ndii mowirikiza bwanji munafuna chithandizo ku sukulu kapena ku ntchito koma sichinapezeke?*	1	2	3	4	5	8		
**g1)** When this problem occurs has it been a big problem or a little problem? *Vuto limeneli likapezeka limakhala lalikulu kapena laling'ono?*							1	2
**h)** In the past 12 months, how often have other people's attitudes toward you been a problem at home? *Pa miyezi khumi ndi iwiri yapitayi, zochitika za anthu ena kwa inu*	1	2	3	4	5	8		

zakhala vuto mowirikiza bwanji kwa inu pa khomo pano?

h1) When this problem occurs has it been a big problem or a little problem? *Vuto limeneli likapezeka limakhala lalikulu kapena laling'ono?* | 1 | 2

i) In the past 12 months, how often have other people's attitudes toward you been a problem at school or work? *Pa miyezi khumi ndi iwiri yapitayi, zochitika za anthu ena kwa inu zakhala vuto mowirikiza bwanji kwa inu ku sukulu kapena ku ntchito?*

1	2	3	4	5	8

i1) When this problem occurs has it been a big problem or a little problem? *Vuto limeneli likapezeka limakhala lalikulu kapena laling'ono?* | 1 | 2

j) In the past 12 months, how often did you experience prejudice or discrimination? *Pa miyezi khumi ndi iwiri yapitayi ndi mowirikiza bwanji mwakumana ndi chitonzo kapena tsankho?*

1	2	3	4	5	8

j1) When this problem occurs has it been a big problem or a little problem? *Vuto limeneli likapezeka limakhala lalikulu kapena laling'ono?* | 1 | 2

Question (35):

Does your usual environment make it easier for you to perform activities of daily living? *[Circle **one** only]*

Pa malo pamene mumakhala anthu amene mumakhala nawo ndi zinthu zina zokuzungulirani, kodi zakuthandizani kuti musavutike kugwira ntchito zanu zatsiku ndi tsiku?

Makes it easier for you:

	Environmental factor	Yes	No	Don't know
a)	Positive attitudes towards you of others<br>*Machitidwe a anthu ena kwa inu*	1	2	8
b)	Support from others when needed<br>*Thandizo lochokera kwa anthu ena mukalifuna*	1	2	8
c)	Accessibility of buildings<br>*Kutha kulowa kapena kutuluka m'nyumba*	1	2	8
d)	Easy terrain (pathways, roads etc.)<br>*Njira kapena mseu wosavuta kuyendamo*	1	2	8
e)	Good climate and other conditions (heat, cold, rain, noise, pollution, no crowding, etc.)<br>*Nyengo yabwinobwino ndi zina monga kutentha, kuzizira, mvula, phokoso, kuonongeka kwa chilengedwe, kusathithikana*	1	2	8
f)	Accessibility of transport<br>*Mayendedwe osavuta*	1	2	8
g)	Service provision	1	2	8

	Kapezedwe ka zisamaliro zosiyanasiyana			
h)	Accessible information<br>*Kapezedwe ka uthenga mosavutikira*	1	2	8
i)	Inclusion in society by all<br>*Kusasalidwa mdera*	1	2	8

SECTION H: HEALTH SERVICE AWARENESS

I am going to ask you about different services:

Tsopano ndikufunsani za chithandizo cha za umoyo chosiyanasiyana

Question (36):
Which services, if any, are you *aware* of and have ever *needed/received*?
[Read out; and enter the appropriate code for each column of each row].

Kodi ndi zithandizo ziti zomwe ndi tchulezi zomwe mukuziwa ndipo munadzifunapo kapena kulandilapo?

			Aware of service **1=Yes** **2=No**	Needed service **1=Yes** **2=No**	Received service **1=Yes** **2=No**
			(1)	(2)	(3)
**a) Medical rehabilitation (*Chithandizo cha kuchipatala)*:**					
	**aa)**	Physiotherapy *(Mafizo/masewera)*	☐	☐	☐
	**ab)**	Occupational therapy *(Kuthandiza anthu kugwira ntchito yodzithandiza atadwala kanthawi*	☐	☐	☐
	**ac)**	Speech therapy (*Kuthandiza munthu kuyankhula)*	☐	☐	☐
	**ad)**	Hearing therapy *(Kuthandiza munthu kumva)*	☐	☐	☐
	**ae)**	Other *(Zina)*	☐	☐	☐
**b) Assistive devices service *(Zipangizo zodzithandizira)*:**					
	**ba)**	Sign language interpreter *(Womasulra zizindikiro za chiyankhulo)*	☐	☐	☐
	**bb)**	Wheelchair *(Njnga)*	☐	☐	☐
	**bc)**	Hearing aids *Zipangizo zothandiza kumva)*	☐	☐	☐
	**bd)**	Visual aids, Braille etc. (*Zipangizo zothandizira kuona/kuwerenga)*	☐	☐	☐
	**be)**	Other *(Zina)*	☐	☐	☐
**c) Primary health care clinic/centre *(Chipatala chaching'ono)***			☐	☐	☐
**d) Secondary health care (e.g. hospital) *(Chipatala chachikulu)***			☐	☐	☐
**e) Mobile health clinic *(Chipatala choyendayenda)***			☐	☐	☐
**f) Home based care *Chisamaliro cha umoyo cha pakhomo)***			☐	☐	☐

**g) Counselling** ***(Uphungu)***			
**ga)** Psychologist *(Dotolo wa maganizo)*	☐	☐	☐
**gb)** Psychiatrist *(Dotolo wa a misala)*	☐	☐	☐
**gc)** Social worker *Mulangizo wa chitukuko)*	☐	☐	☐
**gd)** School counsellor *(Mulangizi wa ku sukulu)*	☐	☐	☐
**ge)** Other *(Zina)*	☐	☐	☐
**h) Health information (*Uthenga wa zaumoyo)***			
**ha)** Media (*Njira zofalitsa uthenga)*	☐	☐	☐
**hb)** Schools *(Masukulu)*	☐	☐	☐
**hc)** Clinics *(Chipatala chaching'ono)*	☐	☐	☐
**hd)** Hospital *(Chipatala chachikulu)*	☐	☐	☐
**he)** Counselling *(Uphungu)*	☐	☐	☐
**hf)** Other *(Zina)*	☐	☐	☐
**i) Traditional healer/faith healer *(Sing'anga)***	☐	☐	☐

SECTION I: HEALTH CARE UTILISATION

I would like to know about your recent experiences with obtaining health care from health care workers, hospitals, clinics and the health care system. I want to know if you needed health care recently, and if so, why you needed health care and what type of health care provider you received care from.

Gawo limeneli tikufuna kudziwazomwe mwakhala mukukumana nazo pofuna chisamaliro cha zaumoyo kuchokera kwa ogwira ntchito za umoyo ndi kuchipatala. Ndifunanso kudziwa ngati mwafuna chisamaliro cha zaumoyo posachedwa, ngati ndi choncho, chifukwa chani mudafuna chisamalirocho ndipo mudachilandira kuchokera kwa ndani.

Question (37):

When was the last time that you needed health care?

Ndi liti munafuna chisamaliro cha za umoyo komaliza?

Years ago	
Months ago	
Weeks ago	
Days ago	
Never	97
Don't know	98

Question (38):

The last time you needed health care, did you get health care?

Nthawi yomaliza imene munafuna thandizo la zaumoyo, munalipeza?

Yes	1	*Skip → Go to Q: 40*

No	2
Don't know/ Don't remember	8

Question (39): Which reason(s) best explains why you did not get health care the last time you needed it? *[Mark all that the respondent indicates, but do not read out items]*

Ndi chifukwa chiti chokwanira chomwe chinakulepheretsani kupeza chisamaliro cha zaumoyo nthawi yomaliza imene munachifuna?

	Reasons	Code
a)	No one to accompany me<br>*Palibe ondiperekeza*	1
b)	Cost<br>*Kusowa ndalama zolipinira*	2
c)	No transport<br>*Kusowa mayendedwe*	3
d)	Discrimination<br>*Tsankho*	4
e)	Attitudes of health care providers<br>*Khalidwe la opereka chithandizo*	5
f)	Had a bad incident and so don't go anymore<br>*Anandipanga chipongwe ndiye ndinasita kupitako*	6
g)	The gender of health care provider<br>*Opereka chithandizo kukhala wankazi kapena wamwamuna*	7
h)	The type (professional category) of health care provider<br>*Udindo wa opereka chithandizo cha mankhwala*	8
i)	Old age<br>*Ukalamba*	9
j)	Disability<br>*Chilema*	10
k)	Crime, danger<br>*Umbanda/kuopsya*	11
l)	Lack of time due to domestic or other Responsibilities<br>*Kusapeza nthawi chifukwa cha zifukwa zina*	12
m)	Lack of medication<br>*Kusowa mankhwala*	13

	Reasons	Code
n)	Did not want family to know I am ill<br>*Sindifuna achibale adziwe matenda anga*	14
o)	There are no services<br>*Kulibe zithandizo*	15
p)	Language barrier<br>*Kusamvana chiyankhulo*	16
q)	Distance from home to clinic<br>*Mtunda wopita ku chipatala*	17
r)	Physical accessibility of the facility<br>*Malo amene chipatala chimapezeka*	18
s)	Not satisfied with outcomes of previous experience<br>*Sindinakhutire ndi chithandizo chomaliza*	19
t)	Traditional (culture)<br>*Chikhalidwe*	20
u)	Opening times are not suitable<br>*Nthawi yotsegulira sili bwino*	21
v)	Not sick enough or not sick (do not need)<br>*Sindinadwale kwambiri*	22
w)	Waiting times too long<br>*Kutalika kwa nthawi yodikira*	23
x)	Religious belief<br>*Chikhulipiliro cha chipembedzo*	24
y)	No knowledge about the health facility<br>*Sindidziwa za chipatala*	25
z)	Other, specify<br>*Zina*	26

Question (40):

Thinking about health care you usually need, where do you normally go? *[Circle **one** only]*

Kodi mukafuna chisamaliro cha za umoyo, mumapita kuti?

Private doctor's office	01
Private clinic or health care facility	02
Private hospital	03
Public clinic or health care facility	04
Public hospital	05
Charity or church run clinic	06
Charity or church run hospital	07
Traditional healer [*use local term*]	08
Faith healer [*use local term*]	09
Pharmacy or dispensary	10
Have not needed any health care in the last 3 years	11
Other (specify)	12

Question (41):

Now, let us think back to the last time you needed health care. What was the reason you needed health care the last time, even if you did not get it?

Kodi ndi chifukwa chani munafuna chisamaliro cha za umoyo ngakhale simunachipeze?

	Office use

Question (42):

The last time you used health care, how did you get there? *[Circle **one** only]*

Nthawi yomaliza kugwiritsa ntchito chisamaliro cha za umoyo mudayenda bwanji kuti mukafikeko?

Private vehicle (car or motorcycle)	1
Public transportation	2
Taxi/cab	3
Bicycle	4
Walk	5
Other (specified)	6
Never used health care	7
Don't know	8

Question (43):

About how long did it take you to get there?

Mudatenga nthawi yayitali bwanji kuti mukafikeko?

Hours	
Minutes	
Don't know	98

Question (44):

Once you got to the health care facility the last time you used health care, how long did you wait?

Mutafika kumalo olandira chithandizo cha za umoyo mudadikira nthawi yayitali bwanji kuti mulandire chisamaliro?

Hours	
Minutes	
Don't know	98

	Yes	No	Don't know/ Don't remember
**Question (45)**: The last time you went to the health facility, did you go with someone to assist you? *Kodi mudapita ndi munthu wokuthandizirani?*	1	2	8
**Question (46)**: Do you usually need someone to come with to assist you whenever you come to the health facility? *Kodi nthawi zambiri mumafuna munthu wokuthandizirani mukamapita ku malo olandira thandizo la za umoyo?*	1	2	8

Question (47):

Considering your own experience, tell me whether the following make it difficult for you to get health care:

[Read out the alternatives, and show card. Circle only ***one*** *code for* ***each*** *row]*

Potengera zomwe mwakumana nazo tandiuzani pa zinthu zomwe nditchulezi ngati zimakukanikitsani kupeza chisamaliro cha za umoyo:

		No problem	Small problem	Moderate problem	Serious problem	Insurmountable problem
**a)**	Lack of transport from home to health facility<br>*Kusowa mayendedwe opitira ku chipatala*	1	2	3	4	5
**b)**	No services available<br>*Kusapezeka kwa chithandizo*	1	2	3	4	5
**c)**	Physical access to facility<br>*Kufilira ku chipatala*	1	2	3	4	5
**d)**	Because of faith/belief<br>*Chikhulupiliro*	1	2	3	4	5
**e)**	Negative attitudes among health workers<br>*Khalidwe loipa la ogwira ntchito*	1	2	3	4	5
**f)**	There is no accommodation at the health facility<br>*Kulibe malo ogona ku chipatala*	1	2	3	4	5
**g)**	Communication with health workers<br>*Kulumikizana ndi opereka chithandizo*	1	2	3	4	5
**h)**	Standard of the health facility<br>*Kawonekedwe ka chipatala*	1	2	3	4	5
**i)**	The journey to the health care is dangerous<br>*Ulendo wopita kuchipatala ndi oopsya*	1	2	3	4	5
**j)**	You did not know where to go<br>*Sindinadziwe kopita*	1	2	3	4	5
**k)**	Could not afford the cost of the visit<br>*Simunakwanitse ndalama zolipira chithandizo*	1	2	3	4	5
**l)**	Don't have the necessary document (health card/passport)<br>*Mulibe zipaso zoyenera (Kabuku ka zaumoyo)*	1	2	3	4	5
**m)**	You thought you were not sick enough<br>*Mumaganiza kuti simunadwale kwambiri*	1	2	3	4	5
**n)**	You tried but were denied health care<br>*Munayesesa koma munakanizidwa chithandizo*	1	2	3	4	5
**o)**	The health care provider's drugs or equipment were inadequate<br>*Zipangizo/mankhwala anali osakwanira*	1	2	3	4	5
**p)**	Could not take time off work or had other commitments<br>*Ndinali otanganidwa ndi ntchito zina*	1	2	3	4	5
**q)**	You were previously badly treated<br>Simunathandizidwe bwino	1	2	3	4	5
**r)**	Could not afford the cost of transport<br>*Munalephera kulipira mayendedwe*	1	2	3	4	5
**s)**	Other (specify)<br>*Zina*	1	2	3	4	5

Question (48):

Considering your experience receiving or visiting health care providers, how would you rate the following? *[Circle **one** only code for each **row**]*

Potengra zomwe mumakumana nazo polandira kapena mukapita kwa opereka chisamaliro cha za umoyo, zosatirazi mungaziike pa mulingo wanji?

		Very good	Good	Moderate	Bad	Very bad
**a)**	The amount of time you waited before being attended to.<br>*Kutalika kwa nthawi yodikilira chithandizo*	1	2	3	4	5
**b)**	Your experience of being treated respectfully.<br>*Ulemu umene munapatsidwa polandira chithandizo*	1	2	3	4	5
**c)**	How clearly health care providers explained things to you.<br>*Mumene opereka chithandizo anafotokozera*	1	2	3	4	5
**d)**	Your experience of being involved in making decisions for your treatment.<br>*Kutengapo kanu mbali pa chithandizo chanu*	1	2	3	4	5
**e)**	The way the health services ensured that you could talk privately to providers.<br>*Njira yimene yinatsatidwa kuti muyankhulane ndi opereka chithandizo panokha*	1	2	3	4	5
**f)**	The ease with which you could see a health care provider you were happy with.<br>*Kuphweka kokumana ndi opereka chithandizo yomwe mumasangalala nayo*	1	2	3	4	5
**g)**	The cleanliness in the health facility.<br>*Ukhondo mu chipatala*	1	2	3	4	5

Question (49):

Overall, how satisfied are you with the provision of health care in your area? *[Circle **one** only]*

Nndinu okhutitsidwa bwanji ndi chisamaliro cha za umoyo chimene chimaperekedwa m'dera lanu?

Very satisfied<br>*Okhutira kwambiri*	1
Satisfied<br>*Okhutira*	2
Neither satisfied nor dissatisfied<br>*Pakatikati*	3
Dissatisfied<br>*Osakhutira*	4
Very dissatisfied<br>*Osakhutira kwambiri*	5

Question (50):

Overall, how satisfied are you with the personnel in your area or those that are accessible to you? *[Circle **one** only]*

Ndinu okhutitsidwa bwanji ndi anthu ogwira ntchito za umoyo m'dera lanu kapena ena omwe mumawafikira?

Very satisfied<br>*Okhutira kwambiri*	1
Satisfied<br>*Okhutira*	2
Neither satisfied nor dissatisfied<br>*Pakatikati*	3
Dissatisfied<br>*Osakhutira*	4
Very dissatisfied<br>*Osakhutira kwambiri*	5

Question (51):

Has a health worker visited you at home during the last 3 years?

Zaka zitatu zapitazo mwayenderedapo ndi ogwira ntchito za umoyo?

Yes	1	
No	2	***Skip → Go to Q: 53***

Question (52):

If yes, how many times? *[Circle **one** only]*

Kangati?

Once	1
A few times	2
Many times	3

Question (53):

Have you ever accessed the following services? *[**Circle ALL that apply** or ONLY 'Not applicable' (8) if none of the services were accessed]*

Kodi munalandirapo zithandizo zotsatirazi?

a) Malaria prevention (bed nets, etc.)	1
b) HIV/AIDS counselling	2
c) HIV/AIDS testing	3
d) Immunisation	4
e) Not applicable	8

SECTION J: ATTITUDES TOWARDS HEALTH SERVICES

Question (54):

Please tell me how much you agree with the following statements. *[Circle only **one** code for each row]*

Pa ziganizo zotsatirazi, mukugwirizana nazo motani?

		**Strongly Disagree**	**Slightly Disagree**	**Slightly Agree**	**Strongly Agree**
**a)**	The health personnel at the local clinic/health center have appropriate competence to help with your health problems<br>*Ogwira ntchito za umoyo pa chipatala chaching'ono ali ndi luso loyenera pothandiza mavuto anu a zaumoyo*	1	2	3	4
**b)**	The health personnel at the hospital have appropriate competence to help with your health problems<br>*Ogwira ntchito za umoyo pa chipatala chachikulu ali ndi luso loyenera pothandiza mavuto anu a za umoyo*	1	2	3	4
**c)**	The traditional healer have appropriate competence to help with your health problems<br>*Asing'anga ali ndi luso loyenera pothandiza mavuto anu a zaumoyo*	1	2	3	4
**d)**	You trust the treatment provided by health personnel at the hospital<br>*Muli ndi chikhulupiliro pa chithandizo chimene mumalandira kwa ogwira ntchito za umoyo pa chipatala*	1	2	3	4
**e)**	You trust the treatment provided by health personnel at the clinic/health center<br>*Muli ndi chikhulupiliro pa chithandizo chimene mumalandira kwa ogwira ntchito za umoyo pa chipatala chancing'ono.*	1	2	3	4
**f)**	You trust the treatment provided by the traditional healer to be effective<br>*Mukukhulupilira kuti chithandizo cha zaumoyo chimene mumalandira kucholera kwa sing'anga kuti ndi chothandiza.*	1	2	3	4
**g)**	People are received in a positive manner in the clinic/health center<br>*Anthu amalandiridwa bwino pa chipatala chaching'ono.*	1	2	3	4
**h)**	People are received in a positive manner at the hospital<br>*Anthu amalandiridwa bwino pa chipatala chachikulu*	1	2	3	4
**i)**	People are received in a positive manner at the traditional healer<br>*Anthu amalandiridwa bwino kwa sing'anga*	1	2	3	4

Question (55):

I would like to ask you how your health has been in general, over the past few weeks.
For the past few weeks have you….? *[Circle **one** per **row**]*

Ndikufuna ndikufunseni momwe moyo wanu wakhalira masabata angapo apitawa:

Masabata angapo apitawa…?

Health in general		Yes	No
**a)**	Been able to concentrate on what you're doing<br>*Kodi mwakhala mukukhazikika m'maganizo pa zomwe mukuchita*	1	2
**b)**	Lost much sleep over worry<br>*Kusowa tulo chifukwa cha nkhawa*	1	2
**c)**	Felt you were playing a useful part in things<br>M'mamva kuti mumatenda mbali yofunikira pa zinthu	1	2
**d)**	Felt capable of making decisions about things<br>*M'mamva kuthekera kupanga ziganizo pa zinthu*	1	2
**e)**	Felt constantly under strain<br>*Kumva kuphinjidwa*	1	2
**f)**	Felt you couldn't overcome your difficulties<br>*M'mamva kuti simungathe kuthana ndi mavuto anu*	1	2
**g)**	Been able to enjoy your normal day-to-day activities<br>*Kusangalitsidwa ndi ntchito zanu za tsiku ndi tsiku*	1	2
**h)**	Been able to face up to your problems<br>*Kuthana ndi mavuto anu*	1	2
**i)**	Been feeling unhappy and depressed<br>*Kukhala wokhumudwa*	1	2
**j)**	Been losing confidence in yourself<br>*Kutaya chikhulupiliromwa iwe mwini*	1	2
**k)**	Been thinking of yourself as a worthless person<br>*Kuziona ngati opanda phindu*	1	2
**l)**	Been feeling reasonably happy, all things considered<br>*Kukhala osangalala*	1	2

Question (56):

Thinking about your general physical health (things like: sickness, illness, injury, disease etc.) – on a scale from 1 (poor) to 4 (very good) – How would you describe your overall physical health today? *[Circle **one** only]*

Mungalongosole bwanji za thanzi lanu la lero?

Poor	1
Not very good	2
Good	3
Very good	4
Don't know	8

Question (57)

Thinking about your general mental health (things like: anxiety, depression, fear, fatigue, tiredness, hopelessness etc.) – on a scale from 1 (poor) to 4 (very good) – How would you describe your overall mental health today? *[Circle **one** only]*

Mungalongosole bwanji za thanzi la m'malingaliro anu?

Poor	1
Not very good	2
Good	3
Very good	4
Don't know	8

Question (58):

Do you consider yourself to have a disability?

Kodi mukuganiza ngati muli ndi chilema?

Yes	1
No	2

Thank you very much for your time!
